# Postoperative SBRT and Severe Late Toxic Effects in Early-Stage Oropharyngeal and Oral Cavity Cancers: The STEREOPOSTOP–GORTEC 2017-03 Nonrandomized Clinical Trial

**DOI:** 10.1001/jamanetworkopen.2025.49975

**Published:** 2025-12-18

**Authors:** Julian Biau, Xushan Sun, Xavier Liem, Jean-Christophe Faivre, Elife Eker, Pierre Blanchard, Yungan Tao, Mélanie Doré, Philippe Maingon, Noémie Vulquin, Marc Alfonsi, Sebastien Thureau, Florence Huguet, Séverine Racadot, Emilie Thivat, Ioana Molnar, Pierre Boisselier, Sébastien Guihard, Olivier Gallocher, Charles Dupin, Stéphanie Batard, Jean Bourhis, Michel Lapeyre

**Affiliations:** 1 Department of Radiation Oncology, Centre Jean Perrin, Clermont-Ferrand, France; 2 INSERM U1240 IMoST, Université Clermont Auvergne, Clermont-Ferrand, France; 3 UMR 501, Centre d’Investigation Clinique, Clermont-Ferrand, F-63001 France; 4 Department of Radiation Oncology, Hôpital Nord Franche-Comté and CHRU Besançon, Montbéliard and Besançon, France; 5 Department of Radiation Oncology, Centre Oscar Lambret, Lille, France; 6 Department of Radiation Oncology, Institut de Cancérologie de Lorraine, Vandoeuvre les Nancy, France; 7 Department of Radiation Oncology, Centre Hospitalier Lyon Sud, Pierre Bénite, France; 8 Department of Radiation Oncology, Gustave Roussy, Villejuif, France; 9 Department of Radiation Oncology, Institut de Cancérologie de l’Ouest, Saint Herblain, France; 10 Department of Radiation Oncology, Groupe Hospitalier La Pitié Salpétrière (AP-HP), Paris, France; 11 Department of Radiation Oncology, Centre Georges François Leclerc, Dijon, France; 12 Department of Radiation Oncology, Institut Sainte Catherine, Avignon, France; 13 Department of Radiation Oncology, Centre Henri Becquerel, Rouen, France; 14 Department of Radiation Oncology, Hôpital Tenon AP-HP, Sorbonne Université, Paris, France; 15 Department of Radiation Oncology, Centre Léon Bérard, Lyon, France; 16 Department of Radiation Oncology, Institut du Cancer de Montpellier, Montpellier, France; 17 Department of Radiation Oncology, ICANS, Strasbourg, France; 18 Department of Radiation Oncology, Clinique Pasteur, Toulouse, France; 19 Department of Radiation Oncology, CHU de Bordeaux, Pessac, France; 20 Department of Radiation Oncology, Institut Bergonié, Bordeaux, France; 21 Department of Radiation Oncology, CHUV, Lausanne, Suisse

## Abstract

**Importance:**

Postoperative radiotherapy (mainly fractionated intensity-modulated radiotherapy) is indicated after surgery for early-stage oral cavity squamous cell carcinoma (OCSCC) and oropharyngeal squamous cell carcinoma (OPSCC) in the presence of high-risk margins. Postoperative brachytherapy is a treatment option, but it is not always feasible; stereotactic body radiotherapy (SBRT) may offer a noninvasive alternative.

**Objective:**

To evaluate late toxic effects and 2-year local control after postoperative SBRT to the primary tumor bed in patients with early-stage OCSCC or OPSCC and high-risk resection margins.

**Design, Setting, and Participants:**

This national, multicenter, single-arm, phase 2 nonrandomized clinical trial was conducted across 18 academic centers in France in the Groupe d’Oncologie Radiothérapie Tête et Cou (GORTEC) network. Adults with pT1 to pT2 OCSCC or OPSCC and R1 or smaller than 5 mm margins, pN0 or pN1 without extracapsular extension, no indication for neck irradiation, and not planned for adjuvant chemotherapy were enrolled between April 2018 and August 2021. Final analysis was conducted from February to May 2024.

**Intervention:**

SBRT to the surgical bed, 36 Gy in 6 fractions over 2 weeks, using volumetric modulated arctherapy (VMAT) or CyberKnife, with image guidance and quality assurance protocols.

**Main Outcomes and Measures:**

The primary outcome was the 2-year rate of grade 3 or greater late toxic effects (>90 days after treatment), per Common Terminology Criteria for Adverse Events version 4.03. The key secondary end point was 2-year local control. Quality of life (QoL) was assessed using European Organization for Research and Treatment of Cancer (EORTC) core Quality of Life Questionnaire (QLQ-C30) and EORTC Quality of Life Questionnaire Head and Neck Module (QLQ-HN35).

**Results:**

Among 90 patients (median [range] age, 64 [31-87] years; 51 [56.5%] male), at 2 years, 2 patients (2.2%) had grade 3 late toxic effects, while 13 patients (14.6%) experienced at least 1 grade 3 late toxic effect during the 2-year follow-up period, mainly soft-tissue necrosis and osteoradionecrosis, most of which were transitory. The 2-year local control rate was 92.0% (95% CI, 84.6%-96.4%). Acute grade 3 or greater toxic effects were limited to mucositis (28.9%; 95% CI, 19.8%-39.4%), mostly resolving by 3 months. Median follow-up was 25 months (range, 3-31 months). Disease-free and overall survival at 2 years were 73.3% (95% CI, 63.4%-81.7%) and 88.8% (95% CI, 80.9%-94.2%), respectively. QoL scores showed a transient decline at 1 month followed by recovery and improvement at 12 and 24 months.

**Conclusions and Relevance:**

In this phase 2 nonrandomized clinical trial, postoperative primary tumor bed SBRT for early-stage OCSCC and OPSCC with high-risk margins was associated with manageable toxic effects and high rates of local control. These findings support SBRT as a promising approach in carefully selected patients, but confirmatory randomized trials are needed.

**Trial Registration:**

ClinicalTrials.gov Identifier: NCT03401840

## Key Points

**Question:**

What late toxic effects and clinical outcomes are associated with postoperative stereotactic body radiotherapy (SBRT) to the primary tumor bed among patients with early-stage oropharyngeal and oral cavity squamous cell carcinomas and high-risk resection margins?

**Findings:**

In this nonrandomized clinical trial including 90 patients, the incidence of severe late toxic effects at 2 years was 2.2%, and the 2-year local control rate was 92.0%. The trial met both predefined efficacy and safety targets.

**Meaning:**

In this nonrandomized clinical trial, postoperative SBRT to the primary tumor bed appeared to be a safe and effective treatment option in this specific population.

## Teaser

This nonrandomized clinical trial evaluates whether postoperative stereotactic body radiotherapy (SBRT), delivered to the tumor bed among patients with early-stage oral cavity and oropharyngeal squamous cell carcinoma and high-risk margins, is associated with toxic effects more than 90 days after treatment.

## Introduction

Early-stage oropharyngeal (OP) and oral cavity (OC) cancers are mainly squamous cell carcinomas (SCC). Their incidence is rising.^1^ Primary surgery is one of the mainstay treatments.^2^ Negative tumor margins greater than 5 mm are recommended.^3,4^ When feasible, re-resection of any high-risk margin is preferred. Otherwise, postoperative radiotherapy (RT) can be indicated.^5,6,7,8^ Both fractionated intensity-modulated RT (IMRT) and brachytherapy can have a role in this setting. Adjuvant postoperative brachytherapy to the primary site for patients with pT1 or pT2 tumors and negative neck dissection is a therapeutic option.^9,10,11,12,13^ Recent Groupe Européen de Curiethérapie–European Society for Radiotherapy and Oncology Advisory Committee for Radiation Oncology Practice recommendations for head and neck brachytherapy stated that adjuvant brachytherapy could be delivered in cases of high-risk features of local failure, even for positive margins, in selected cases.^13^ Brachytherapy is a highly conformal RT technique that delivers high doses to small volumes within a short overall treatment time. However, brachytherapy is not always technically possible and requires a highly experienced team and appropriate infrastructure. A possible alternative could be postoperative stereotactic body RT (SBRT), which has been investigated in the STEREOPOSTOP trial.^14^ To date, SBRT in head and neck cancers has been mainly studied in cases of reirradiation or as a boost for patients who did not undergo operations.^15,16,17,18,19,20,21,22^ SBRT is an attractive option because it delivers a highly conformal dose of RT in a limited number of fractions (similar to brachytherapy but with fewer technical limitations) with steep dose gradients, resulting in reduced normal tissue irradiation and with a short overall treatment time. To our knowledge, STEREOPOSTOP is the first trial to evaluate postoperative SBRT to the tumor bed in this specific indication of early-stage OCSCC and OPSCC with high-risk margins.

## Methods

### Study Design and Participants

This was as a national, open-label, nonrandomized phase 2 clinical trial. Patients were recruited from 18 hospitals of the Groupe d’Oncologie Radiothérapie Tête et Cou (GORTEC) network in France. The detailed protocol has been published previously^14^ and appears in Supplement 1. The key eligibility criteria were age 18 years or older, Eastern Cooperative Oncology Group (ECOG) status of 2 or less, resected OCSCC (except lips) or OPSCC, pT1 or pT2 (American Joint Committee on Cancer*, *seventh edition), with an indication of postoperative primary tumor bed irradiation (confirmed by multidisciplinary tumor board [MTD]) with at least 1 of the following criteria: positive R1 resection margin (re-resection not proposed), margin of less than 5 mm (re-resection not proposed) or margin estimated at risk with uncertain pathological margin, pN0 after surgical treatment of the neck (lymph node dissection or sentinel lymph node biopsy) or pN1 without extracapsular extension (lymph node dissection). Key exclusion criteria were histology other than SCC; pT3 or pT4; pT2 >3 cm and R1 with concurrent chemoradiotherapy decided in MTD; lymphovascular invasion requiring neck irradiation; neck irradiation decided in MTD; prior RT to the head and neck area; and distant metastasis. Oral and dental examination was mandatory. When indicated, extraction of dental elements was carried out. Adequate dental care (including daily fluorine application if necessary) was recommended to all patients, at least during follow-up.

The study underwent independent human ethics review board approval at CPP Ile de France VI and was registered. All patients provided written informed consent before any study-related assessment. The trial was performed in accordance with the principles of the Declaration of Helsinki, the Good Clinical Practice guidelines of the International Conference on Harmonisation, and local regulatory requirements.

### Procedures

The intervention was a 6-fraction of 6 Gy SBRT for a total dose of 36 Gy to the primary tumor bed. All patients were irradiated in a supine position. Immobilization devices, such as stereotactic customized masks, were mandatory. Planning computed tomography (CT), using a set of slices (1.00-1.25 mm) extending from the level of the base of skull to the lower border of the clavicle, was required. The use of intravenous contrast enhancement was mandatory (except in case of a contraindication). The clinical target volume (CTV) corresponded to the initial tumor bed including the positive or close margins with a margin from 5 to 10 mm according to the anatomical barriers and the spread zones. In case of flap reconstruction, CTV also included the junction of the normal tissue–flap plus a 5-mm proximity flap. A set-up margin was implemented around each CTV to obtain the planning target volume (PTV) taking into account patient set-up uncertainties. Typically, for patients immobilized with a stereotactic device, a 2-mm margin appeared adequate. The delineation included different organs at risk (OAR) according to Brouwer et al.^23^ Either volumetric modulated arctherapy (VMAT) or Cyberknife (CK) SBRT treatments were allowed. The total dose of 36 Gy in 6 fractions was prescribed every other day. Prescription isodose lines were chosen according to treatment facilities, for 36 Gy (100% isodose) to at least encompass 95% or more of the PTV, with no more than 5% of the PTV receiving more than 110% of the prescribed dose (39.6 Gy) for VMAT, and no more than 20% for CK. Online review of the optimal patient repositioning system was mandatory before each fraction. A maximal time interval between surgery and SBRT of 6 weeks was recommended.

Participating centers were required to undergo a comprehensive educational and quality assurance process that included detailed written instructions, and a standardized benchmark case both for delineation and SBRT planning. A retrospective central review of all cases was done.

Three visits with a physical evaluation took place during radiotherapy. After SBRT treatment, a visit was planned 2 weeks after the last fraction, at 1 month, at 3 months, and then every 3 months during the 2 years following SBRT. A head, neck, and chest CT scan was performed at 3 months and at 1 and 2 years. Magnetic resonance imaging and/or fluorodeoxyglucose–positron emission tomography imaging assessments were left to the discretion of the investigators at each center.

### Outcomes

The primary end point was 2-year severe (grade ≥3; >90 days after starting SBRT) toxic effects considered related to postoperative SBRT, assessed according to National Cancer Institute (NCI) Common Terminology Criteria for Adverse Events (CTCAE) version 4.03. Secondary end points included local control (any local recurrence documented in the area of the primary tumor bed); locoregional control (any local or lymph node recurrence); acute toxic effects (within 90 days of starting SBRT) according to NCI CTCAE version 4.03; disease-free survival (DFS, defined as time from enrollment to the date of first occurrence of any locoregional recurrence, distant progression, or death from any cause); overall survival (OS, defined as time from enrollment to death from any cause); quality of life (QoL) of patients (evaluated by the European Organization for Research and Treatment of Cancer [EORTC] core Quality of Life Questionnaire EORTC [QLQ-C30] and EORTC Quality of Life Questionnaire Head and Neck Module [QLQ-HN35] at inclusion, 1 month, 1 year, and 2 years after SBRT).

### Statistical Analysis

Sample size was determined using Fleming single-stage design, taking into account 2 objectives: to assess the 2-year incidence of grade 3 or greater toxic effects (primary end point) and to evaluate the 2-year local control rate (key secondary end point). For late toxic effects, we defined a rate less than 5% as acceptable and greater than 15% as unacceptable (1-sided α = .05, β = 0.10). For local control, we targeted a 2-year rate greater than 90% and considered less than 80% unacceptable (1-sided α = .05, β = 0.20). Allowing for potential withdrawal, the total sample size was set at 90 patients. All end points were assessed in the intention-to-treat (ITT) population. The follow-up time was calculated from enrollment to the date of the last follow-up. Categorical variables were summarized as frequencies and percentages, with 95% CIs. Continuous variables were described using means and SDs. Local control, locoregional control, DFS, and OS were estimated using Kaplan-Meier’s method, with 95% CI derived from the Rothman method. In addition to Kaplan-Meier estimates at fixed time points, we summarized time-to-event outcomes using the restricted mean survival time (RMST). An analysis of factors associated with outcomes was conducted to identify clinical and dosimetric factors associated with differences in 2-year severe toxic effects, local control, and regional control. In a multivariate context, these factors were evaluated by a backward and forward stepwise approach applied to a logistic regression model. The longitudinal analysis of QoL variations was tested by random-effects models, useful for considering between- and within-patient variability. Because this trial was exploratory, no type I error correction was applied for multiple comparisons. Tests were 2-sided, and *P* < .05 was considered significant. A sensitivity analysis was performed to measure the possible impact of missing data and to propose the most appropriate imputation approach. Analyses were conducted with R version 4.3.0 (R Project for Statistical Computing) from February to May 2024.

## Results

**Figure 1.**
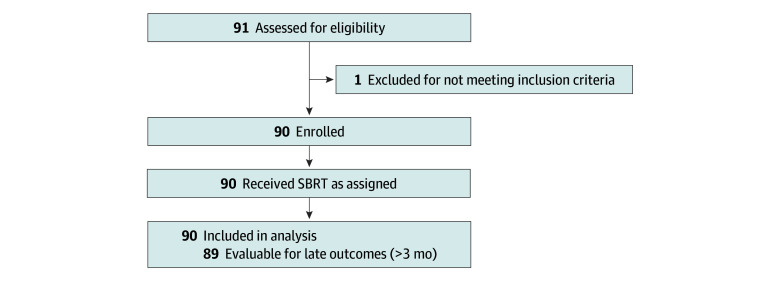
Study Flow Diagram

Between April 2018 and August 2021, 90 patients were included (Figure 1). Baseline patient and tumor characteristics are described in Table 1. There were 51 male (56.5%) and 39 female (43.5%) patients. The median (range) age was 64 (31-87) years. Overall, 74 patients (82.2%) had OCSCC, and 16 (17.8%) had OPSCC. There were 35 patients (38.9%) with pT1 tumors and 55 (61.1%) with pT2 tumors. All but 1 patient were pN0. A total of 9 patients (10.0%) had a sentinel lymph node surgery, and 81 patients (90.0%) a neck dissection, with a median (range) number of analyzed lymph nodes of 21 (6-55). The indications of postoperative SBRT were as follows: 27 (30%) positive R1 margin, 58 (64.4%) close margin of less than 5 mm, and 5 (5.6%) margins estimated at risk. Overall, 68 patients (76%) were treated using VMAT and 22 (24%) using CK. All patients received the entire number of fractions. Median (range) overall treatment time was 11 (9-19) days. The median (range) time from surgery to the start of SBRT was 48 (27-98) days. Median (range) follow-up was 25 (3-31) months. No patient was lost to follow-up. There were no missing data concerning the primary end point and key secondary end point.

**Table 1.** Baseline Patient and SBRT Characteristics

Characteristic	Patients, No. (%)
Age, median (range), y	64 (31-87)
Sex	
Male	51 (56.5)
Female	39 (43.5)
Smoking status	
Never	18 (20.0)
Former	39 (44.0)
Current	32 (36.0)
ECOG status	
0	54 (60.0)
1	31 (34.4)
2	5 (5.6)
Tumor location	
Oral cavity	74 (82.2)
Mobile tongue	38 (42.2)
Floor of mouth	30 (33.3)
Other	6 (6.7)
Oropharynx	16 (17.8)
Tonsil	10 (11.1)
Other	6 (6.7)
T status	
pT1	35 (38.9)
pT2	55 (61.1)
Margin	
R1, <1 mm	27 (30.0)
Close, ≥1 to <5 mm	58 (64.4)
Estimated at risk	5 (5.6)
Perineural invasion	
Yes	26 (29)
No	64 (71)
Nodal surgery	
Lymphadenectomy	81 (90.0)
Analyzed nodes, median (range), No.	21 (6-55)
Sentinel lymph node	9 (10.0)
N status	
pN0	89 (98.9)
pN1	1 (1.1)
SBRT technique	
VMAT	68 (75.6)
CK	22 (24.4)
No. of fractions	
6	90 (100)
SBRT treatment time, median (range), d	11 (9-19)
Time from surgery to SBRT, median (range), d	48 (27-98)

Abbreviations: CK, CyberKnife; ECOG, Eastern Cooperative Oncology Group; SBRT, stereotactic body radiotherapy; VMAT, volumetric modulated arctherapy.

Patients’ late toxic effects (primary end point) are summarized in Table 2. Of the 90 enrolled patients, 89 (98.9%) were evaluable for late toxic effects. One patient experienced an early local recurrence at 3 months after SBRT and was therefore excluded from late toxic effects assessment. There were no grade 4 or greater radio-induced late toxic effects. At 2 years, 2 patients (2.2%; 95% CI, 0.3%-7.8%) had an ongoing grade 3 late toxic effect, while 13 patients (14.6%; 95% CI, 8.1%-23.7%) experienced at least 1 grade 3 late toxic effect during the 2-year follow-up period, most of which were transitory. This met the predefined threshold for the primary end point. Overall, 4 patients (4.5%; 95% CI, 1.2%-11.2%) and 3 patients (3.4%; 95% CI, 0.7%-9.6%) experienced transitory grade 3 mucositis and soft-tissue necrosis, respectively, all resolutive between 1 and 8 months. A total of 7 patients (7.9%; 95% CI, 3.2%-15.6%) experienced grade 3 osteoradionecrosis (ORN), of which 6 resolved within 1 to 5 months (1 case unknown). There were no statistically significant clinical or dosimetric parameters associated with late grade 3 toxic effects. However, a higher incidence of ORN was observed in patients treated with CK (18.2%; 95% CI, 5.2%-40.3%) than with VMAT (3.8%; 95% CI, 0.9%-12.4%), but the difference was not statistically significant (*P* = .06). The volume of mandible receiving 29 Gy or higher (V29) and 32 Gy or higher (V32) was higher in patients with grade 3 ORN vs those without (mean [SD] V29, 21.2% [15.2] vs 13.15% [10.2]; mean [SD] V32, 16.9% [14.6] vs 9.6% [8.7]), but these differences were not statistically significant (eTable in Supplement 2).

**Table 2.** Late Grade 3 Toxic Effects^a^

Toxic effect	Patients, No. (%)	Onset, median (range), mo after treatment	Resolution rate, No./total No. (%)	Time to resolution, median (range), mo	Patients with adverse toxic effect at 2 y, No. (%)
Osteoradionecrosis	7 (7.9)	15 (6-24)	6/7 (86)	4.5 (1-5)	1 (1.1)
Soft-tissue necrosis	3 (3.4)	6 (6-6)	3/3 (100)	4.5 (3-8)	0
Mucositis	4 (4.5)	6 (3-18)	4/4 (100)	2.5 (1-8)	0
Pain	2 (2.2)	NA (6-9)	2/2 (100)	NA (3-4)	0
Dysphagia	1 (1.1)	24	NA	NA	1 (1.1)

Abbreviation: NA, not applicable.

^a^ Among 89 of 90 evaluated patients. Adverse events were graded according to Common Terminology Criteria for Adverse Events version 4.03. No grade 4 treatment-related acute toxic effects or deaths occurred.

Patients’ acute toxic effects are summarized in Table 3. All patients were evaluable for acute toxic effects. There was no grade 4 or greater acute toxic effect. The most common acute toxic effect was mucositis, with 55.5% (95% CI, 44.7%-66.1%) and 28.9% (95% CI, 19.8%-39.4%) grade 2 and 3, respectively. At the end of SBRT, 51.1% of patients had grade 2 or 3 mucositis, 75.6% 2 weeks after, 23.3% at 1 month, and 5.5% at 3 months. Other grade 3 acute toxic effects were dysphagia in 5.5% (95% CI, 1.8%-12.5%) of patients, pain in 3.5% (95% CI, 0.7%-9.4%), epidermitis in 1.1% (95% CI, 0-6.0%), and dysgeusia in 1.1% (95% CI, 0-6.0%).

**Table 3.** Acute Toxic Effects^a^

Toxic effect	Patients, by grade of toxic effect, No. (%)		
	Grade 1	Grade 2	Grade 3
Mucositis	9 (10.0)	50 (55.6)	26 (28.9)
Pain	22 (24.5)	17 (18.9)	3 (3.3)
Xerostomia	44 (48.9)	7 (7.8)	0
Dysphagia	21 (23.3)	28 (31.1)	5 (5.6)
Epidermitis	26 (28.9)	3 (3.3)	1 (1.1)
Dysgueusia	18 (20.0)	11 (12.2)	1 (1.1)
Cheilitis	2 (2.2)	6 (6.7)	0
Edema	5 (5.6)	2 (2.2)	0
Trismus	1 (1.1)	1 (1.1)	0

^a^ Among 90 of 90 evaluated patients. Adverse events were graded according to Common Terminology Criteria for Adverse Events version 4.03. No grade 4 treatment-related acute toxic effects or deaths occurred.

**Figure 2.**
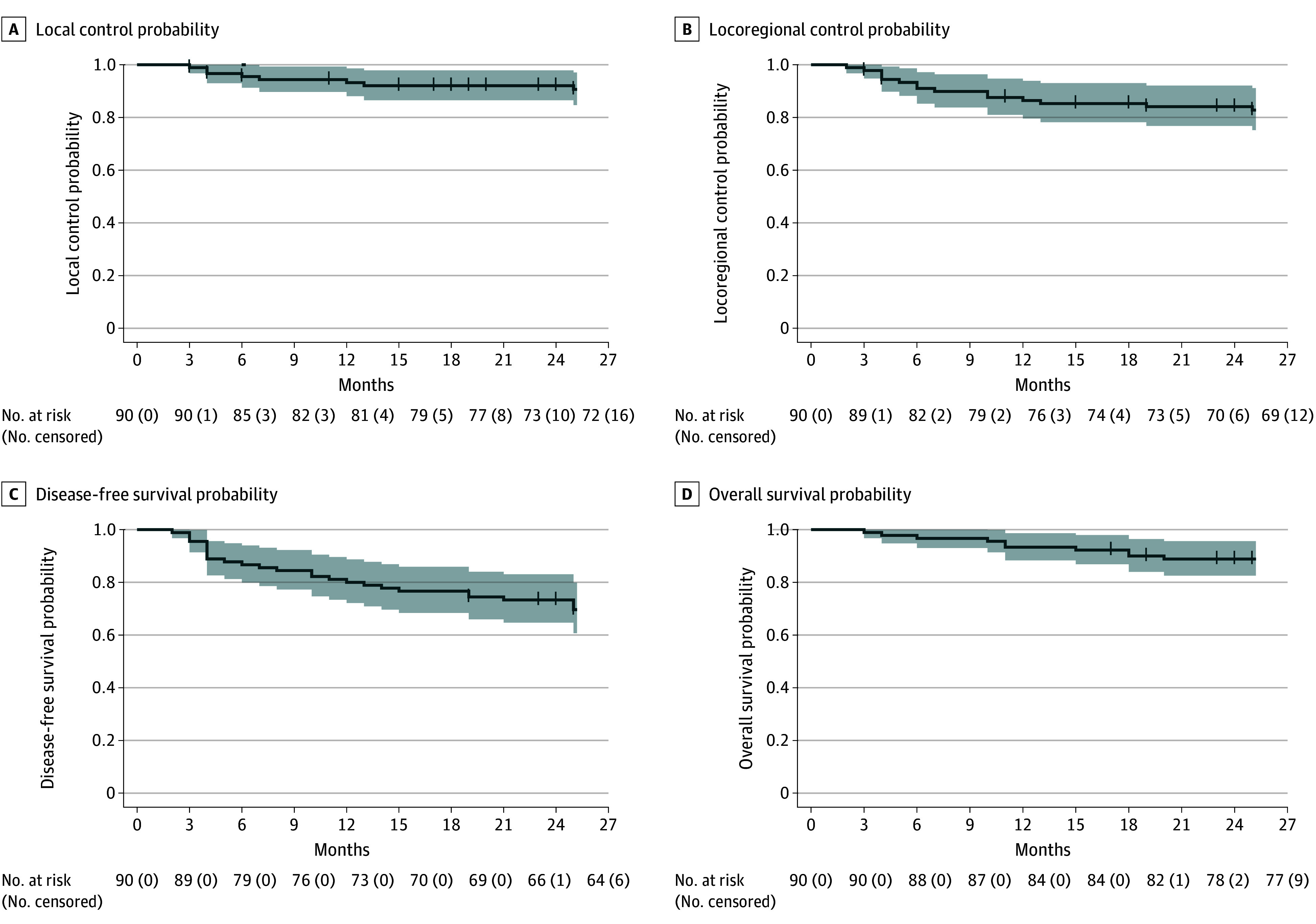
Kaplan-Meier Curves for Local Control, Locoregional Control, Disease-Free Survival, and Overall Survival Shaded areas indicate 95% CIs.

Overall, 8 patients (8.9%) had a local recurrence, of whom 2 (2.2%) had both a local and regional recurrence. Local control rates at 6, 12, and 24 months were 95.5% (95% CI, 89.5%-98.6%), 93.2% (95% CI, 86.3%-97.2%), and 92.0% (95% CI, 84.6%-96.4%) respectively (Figure 2A). This met the predefined threshold for the key secondary end point. For pT1 tumors, 1 of 35 (2.9%) had a local recurrence compared with 7 of 55 with pT2 tumors (12.7%) (*P* = .14). At 24 months, the RMST for local control was 22.6 months (95% CI, 21.7-23.6 months). Of the 8 local recurrences, 3 (37.5%) occurred in patients with positive R1 margins and 5 (62.5%) in those non-R1. This corresponds to 11.1% (95% CI, 2.4%-29.2% [3 of 27]) for R1 and 8.6% (95% CI, 2.9%-19.0% [5 of 58]) for non-R1, with no statistically significant difference (*P* = .70). Overall, 7 patients (7.8%) had a regional recurrence without local recurrence. These 7 patients all had a lymph node dissection with 10 analyzed lymph nodes for 2 of them, and more than 18 for the 5 others. Locoregional control rates at 6, 12, and 24 months were 91.0% (95% CI, 83.6%-95.7%), 86.4% (95% CI, 78.0%-92.4%), and 84.1% (95% CI, 75.1%-90.6%), respectively (Figure 2B). At 24 months, the RMST for locoregional control was 21.4 months (95% CI, 20.1-22.7 months). A total of 7 patients (7.8%) had a distant recurrence, and 7 patients (7.8%) had a second cancer during follow-up.

At time of study analysis, 10 patients (11.1%) had died. Three of these 10 deaths (33.3%) were due to the initially treated cancers. DFS rates at 6, 12, and 24 months were 86.7% (95% CI, 78.4%-92.6%), 80.0% (95% CI, 70.8%-87.3%), and 73.3% (95% CI, 63.4%-81.7%), respectively (Figure 2C). At 24 months, the RMST for DFS was 19.9 months (95% CI, 18.3-21.4 months). OS rates at 6, 12, and 24 months were 96.7% (95% CI, 91.2%-99.1%), 93.3% (95% CI, 86.7%-97.3%), and 88.8% (95% CI, 80.9%-94.2%), respectively (Figure 2D). At 24 months, the RMST for OS was 22.6 months (95% CI, 21.7-23.5 months).

Concerning QoL, there was a statistical difference between scores during follow-up. Globally, QoL worsened between inclusion and 1 month after SBRT and then improved at 12 and 24 months. All data are presented in the eFigure in Supplement 2. The QLQ-C30 Functional score and Global Health Status slightly decreased at 1 month (mean scores, 78 and 62, respectively) compared with baseline (mean scores, 81 and 66, respectively), followed by an improvement at 12 months (mean scores, 82 and 71, respectively) and 24 months (mean scores, 85 and 73, respectively). The QLQ-C30 Symptom score peaked at 1 month (mean, 24), before decreasing below baseline levels at 12 (mean, 17) and 24 months (mean, 14). Similarly, the QLQ-HN35 score, which captures head and neck cancer–specific symptoms, showed a peak at 1 month (mean, 33), and a gradual return to lower levels at 12 (mean, 21) and 24 months (mean, 15), indicating long-term symptom resolution in most patients.

## Discussion

To our knowledge, the STEREOPOSTOP trial^14^ is the first phase 2 trial to evaluate postoperative SBRT to the primary tumor bed for early-stage OCSCC and OPSCC with high-risk margins. The primary end point was met with 2.3% of patients experiencing 2-year grade 3 late toxic effects, and 13.6% of patients experiencing grade 3 late toxic effects within 2 years, most of which were transitory. A total of 5 patients (5.6%) experienced transitory grade 3 soft-tissue necrosis; and 7 patients (7.8%) experienced grade 3 ORN. This toxicity profile seems comparable with the one reported in the series of postoperative brachytherapy published by Strnad et al^24^ in 2013 with 385 patients, which to our knowledge is the largest brachytherapy series worldwide. Brachytherapy was preceded by surgery in 85% of patients. Patients were treated with interstitial pulsed-dose-rate (PDR) brachytherapy. Soft-tissue necrosis and ORN were observed in 10.2% and 4.9% of patients, respectively. Goineau et al^12^ published in 2015 a retrospective series of 112 patients treated with postoperative interstitial low-dose rate (LDR) ^192^Ir brachytherapy for mobile tongue SCC. In this series, grade 2 or greater tongue necrosis requiring surgery was experienced by 22% of patients (mean time from brachytherapy to surgery, 20 months). Few series assessed late toxic effects after conventionally fractionated postoperative RT for early stage head and neck SCC. In 2017, Pedro et al^25^ published a retrospective series of 61 patients with early-stage OPSCC.^25^ Of them, 42 (69%) had a surgical resection, of whom 37 (88%) had adjuvant RT. Severe late toxic effects were reported in 6 patients (11%), all of whom had grade 4 ORN. Recently, a systemic review and meta-analysis^26^ assessed the incidence of ORN of the jaws in patients with oral cancer treated with IMRT. Of a total sample of 1434 patients, 10.4% developed ORN. The appearance of these cases occurred at a mean time of 31.7 months. We observed more grade 3 ORN for patients treated with CK than with VMAT, although the difference was not statistically significant (18% vs 4%; *P* = .06), and higher V29 and V32 to the mandible (not statistically significant, but few events [n = 7]).

The acute toxicity profile that we reported here also appears favorable. The most common acute toxic effects that we reported were grade 2 (55.5%) to grade 3 (28.9%) acute mucositis. Most cases of mucositis were resolutive, with only 5% reporting 3-month grade 2 or 3 mucositis. As noted by Strnad et al,^11^ after brachytherapy, nearly all patients showed a mucositis grade 2 to grade 3 in the implanted area.

Concerning local control, 8 patients (8.9%) had a local recurrence. Local control rates at 6, 12, and 24 months were 100%, 93%, and 92%, respectively. The 2-year local rate control rate of 92% was greater than the predefined targeted rate for the key secondary end point. Postoperative brachytherapy series in this setting report similar local control rates.^12,24,27^ In the series by Lapeyre et al^27^ reporting the outcomes of 36 patients with pT1 to pT2 OCSCC with close or positive margins treated with postoperative interstitial ^192^Ir brachytherapy, the 24-month local control rate was 88.5%. In the series by Mattei et al^28^ including 79 patients with early-stage OCSCC treated with adjuvant LDR- or PDR-brachytherapy to the tumor bed as the sole adjuvant treatment, the 1- and 3-year local control rates were 93.3% and 78.3%, respectively. An additional point concerns the possible inclusion of patients with positive margins (R1) in small tumors (pT1 and pT2 <3 cm) in our trial. According to current international guidelines, concomitant chemoradiotherapy is usually recommended in this situation. However, brachytherapy guidelines still acknowledge the possibility of local radiation treatment alone in selected cases.^13^ In our trial, this option was retained, as in all brachytherapy series.^9,10,11,12,13^ We did not find any statistically significant difference according to margin status. Given the small subgroup sizes, these estimates should be interpreted cautiously and viewed as hypothesis generating.

Our study proposed adjuvant postoperative local SBRT treatment alone, without neck irradiation. All patients but 1 were pN0. Seven patients (7.8%) had a regional recurrence without local recurrence. In 2020, an equivalence randomized trial compared sentinel node biopsy vs lymph node dissection in resectable T1 to T2N0 oral and oropharyngeal cancer.^29^ Data from 279 patients were analyzed, among whom 212 (76%) did not receive adjuvant irradiation and thus did not undergo adjuvant neck irradiation, similar to our study. The presence of neck node recurrence without relapse of the primary tumor was observed in 27 patients (9.7%). Similar rates were reported by D’Cruz et al,^30^ which randomized elective vs therapeutic neck dissection for early stage node-negative oral cancer. Of the 243 patients randomized in the elective neck dissection group, 25 (10.3%) experienced an isolated nodal relapse.

In this study, we also assessed QoL of patients. Although a transient deterioration was observed at 1 month after treatment, these changes were largely reversible. Between baseline and 24 months, all QoL scores improved, with the QLQ-HN35 showing an improvement of more than 10 points, supporting a clinically meaningful reduction in symptom burden over time.^31,32^ These findings are consistent with previous reports describing a U-shaped QoL trajectory among patients with head and neck cancer.^33,34,35^

### Limitations

This study has limitations. This single-arm phase 2 nonrandomized trial was exploratory and does not supplant the current standard of care. Our study allowed the inclusion of patients with R1 margins, for which postoperative chemoradiotherapy remains recommended. Our results should not be interpreted as challenging these standards. Instead, they provide preliminary evidence that postoperative SBRT might be feasible in specific circumstances when alternative conformal local treatments, such as brachytherapy, were previously studied. Larger randomized clinical trials with longer follow-up are required before considering broader clinical adoption.

Another limitation relates to the reporting of ORN. Although 7 patients (7.8%) developed grade 3 ORN, detailed data on the management of these events were not systematically collected. Available information suggests that most cases were transient, but we cannot fully assess the proportion requiring invasive interventions. Future studies should incorporate standardized reporting of ORN management and outcomes to better capture its true burden. Another limitation of our study is the relatively short follow-up, with a median of 25 months. While historically the first 2 years after RT have been considered the most critical period for the development of ORN, more recent data suggest that late cases continue to occur beyond this timeframe.

## Conclusions

In this phase 2 nonrandomized trial of postoperative tumor bed SBRT for adults with pT1 or pT2 oropharyngeal and oral cavity cancers at risk of local recurrence, there were few late toxic effects and rates of 2-year local recurrence were low. These findings support SBRT as a promising approach in carefully selected patients, but confirmatory randomized clinical trials are needed.

## Notes

Supplement 1.
Trial Protocol.

Supplement 2. **eTable.** Mandibular Dose-Volume Indices and Osteoradionecrosis Risk **eFigure.** Evolution of Mean Quality Of Life Scores Over Time, Assessed Using the EORTC QLQ-C30 and QLQ-HN35 Questionnaires

Supplement 3.
Data Sharing Statement
