# Supplementary material for: Postoperative SBRT and Severe Late Toxic Effects in Early-Stage Oropharyngeal and Oral Cavity Cancers: The STEREOPOSTOP–GORTEC 2017-03 Nonrandomized Clinical Trial
Source: JAMA Netw Open. 2025 Dec 18;8(12):e2549975. doi: 10.1001/jamanetworkopen.2025.49975 (PMC12715650; doi:10.1001/jamanetworkopen.2025.49975)
Supplement: Supplement 1. — Trial Protocol. [file jamanetwopen-e2549975-s001.pdf]

GORTEC Protocol 2017 - 03 : STEREO - POSTOP

MULTICENTER PHASE II STUDY OF POSTOPERATIVE HYPOFRACTIONATED STEREOTACTIC RADIOTHERAPY OF EARLY STAGE CANCERS OF THE OROPHARYNX AND ORAL CAVITY WITH HIGH RISK MARGINS.

Short title: STEREO – POSTOP *(Postoperative Stereotaxy*)

Version:4.0 **As of: July 27, 2020**

ID - RCB 2017-A02058-45

Sponsor Code GORTEC 2017 – 03

Promoter
Centre Jean Perrin
58 Rue de Montalembert
63003 Clermont-Ferrand Cedex 1

Partner
GORTEC (Head and Neck Radiotherapy Oncology Group)
Hôpital Bretonneau, 2 Boulevard Tonnellé, 37044 Tours
Tel.: +33 9 67 27 89 51 Fax: +33 2 47 72 89 51
Website: http://www.gortec.fr/

Investigators Coordinators

Dr Julian BIAU
Department of Radiotherapy
Centre Jean Perrin, 58, rue Montalembert
63011 Clermont-Ferrand
Phone : 04 73 27 81 42
Fax: 04 73 27 81 25
@: julian.biau@clermont.unicancer.fr

Dr Michel LAPEYRE
Department of Radiotherapy
Centre Jean Perrin, 58, rue Montalembert 63011 Clermont-Ferrand
Phone : 04 73 27 81 42
Fax: 04 73 27 81 25
@ : michel.lapeyre@clermont.unicancer.fr

Intergroup Coordinator

Prof. Jean BOURHIS, President
Head of the Radiation Oncology Department, CHUV
Hospital building, Rue du Bugnon 46
CH-1011 Lausanne, Switzerland
Tel: +41 21 314 46 65 (secretariat)
Fax: +41 (0)21 314 46 01
@: jean.bourhis@chuv.ch

PROTOCOL APPROVAL

Multicenter phase II study of postoperative hypofractionated stereotactic radiotherapy of early stage cancers of the oropharynx and oral cavity with high risk margins.

GORTEC 2017 – 03: STEREO-POSTOP

Version 3.0 November 25, 2019

RCB ID: 2017-A02058-45

AUTHORIZATION/NOTICE	DATE	REFERENCE
ANSM	25/12/2017	BCRDI: 2017-A02058-45
CPP	01/12/2017	67-17

This version of the protocol is approved by:

Principal Coordinating Investigator: **Dr Julian BIAU**<br><br>**Date.................** **Signature:..................**
Principal Co-Coordinator Investigator: **Dr Michel LAPEYRE**<br><br>**Date.................** **Signature:..................**

I, the undersigned, Doctor / Professor: ....
(Principal Investigator)

Investigator: ....
(Name of the institution, Department)

After reading this protocol, certify that I will conduct this study according to European rules, in accordance with the Declaration of Helsinki, the Jardé law and the principles of Good Clinical Practice.

I am committed to:

- To obtain from each patient his or her consent to participate in the study, given of his or her own free will, after having made him or her aware of the information letter intended for the patient;
- To proceed with the reporting of all serious adverse events as provided for in the protocol;
- To meet the inclusion and non-inclusion criteria as well as the start and end dates of the study;
- To fill in all the sections of the report book;
- To respond to requests for rectifications or clarifications in connection with the observation form;
- To accept regular check-ups;
- To archive and retain the trial documents for 15 years.

Date : **Signature:**

STUDY CONTACTS

**Promoter**	**JEAN PERRIN CENTER**<br>58, rue Montalembert, 63011 CLERMONT-FERRAND<br>Phone : 04 73 27 80 80 - Fax : 04 73 26 34 51	
**Partner**	**GORTEC** *(Head and Neck Radiotherapy Oncology Group)*<br>Hôpital Bretonneau, 2 Boulevard Tonnellé, 37044 Tours<br>Tel.: 09 67 27 89 51 - Fax: 02 47 72 89 51<br>Website: http://www.gortec.fr/	
**Coordinating Investigators**	**PRINCIPAL INVESTIGATOR**<br>**Dr. Julian BIAU**<br>Department of Radiotherapy,<br>Centre Jean Perrin, 58, rue Montalembert<br>63011 Clermont-Ferrand<br>Phone : 04 73 27 81 42 - Fax : 04 73 27 81 25<br>@ julian.biau@clermont.unicancer.fr	**CO-PRINCIPAL INVESTIGATOR**<br>**Dr. Michel LAPEYRE**<br>Department of Radiotherapy,<br>Centre Jean Perrin, 58, rue Montalembert<br>63011 Clermont-Ferrand<br>Phone : 04 73 27 81 42 - Fax : 04 73 27 81 25<br>@ michel.lapeyre@clermont.unicancer.fr
	**ENT INTERGROUP – GORTEC**<br>**Prof. Jean BOURHIS, President**<br>Head of the Radiation Oncology Department, CHUV<br>Hospital Building, Rue du Bugnon 46, CH-1011 Lausanne, Switzerland<br>Tel: +41 21 314 46 65 - Fax: +41 (0)21 314 46 01<br>@ jean.bourhis@chuv.ch	
**Project Managers**	**JEAN PERRIN CENTER**<br>**Mrs. Emilie THIVAT**<br>*Jean Perrin Centre,*<br>*58, rue Montalembert*<br>*63011 Clermont-Ferrand*<br>Tel: 04 73 27 80 89 - Fax: 04 73 27 80 29<br>@ emilie.thivat@clermont.unicancer.fr	**GORTEC**<br>**M. Martial BINDZI**<br>Bretonneau Hospital,<br>2 Boulevard Tonnellé, 37044 Tours<br>Tel.: 02 42 06 01 87 Fax: 02 47 77 05 4802 42 06 01 76<br>@ martial.bindzi@gortec.fr
**Data-management & Biostatistics**	**DATA MANAGER**<br>**Mr. Laurent GIBEL**<br>Bretonneau Hospital - 2, Boulevard Tonnellé<br>37044 Tours, France<br>Tel: 02 47 31 01 88 - Fax: 02 47 77 05 48<br>@ laurent.gibel@gortec.fr	**BIOSTATISTICIANS**<br>**Ms. Ioana MOLNAR**<br>Phone : 04 73 27 80 75<br>ioana.molnar@clermont.unicancer.fr<br>**Mr. Bruno PEREIRA**<br>bpereira@chu-clermontferrand.fr<br>Tel: 04 73 17 84 10
**Pharmacovigilance**	**Service de Pharmacovigilance Centre Jean Perrin**<br>**Sophie LEVESQUE**<br>**Fax: 04 73 27 80 29**<br>@ sophie.levesque@clermont.unicancer.fr	
**Head of the Radiotherapy Quality Assurance Committee (AQ-RT)**	**Dr. Yungan TAO**<br>Department of Radiotherapy<br>Gustave Roussy<br>Tel: +33 1 42 11 65 32 - Fax: +33 1 42 11 52 53<br>@ yungan.tao@gustaveroussy.fr	

**Protocol Review Committee**	**Dr Séverine Racadot.** Léon Bérard Centre, Lyon<br>**Dr. Florence Huguet.** Tenon Hospital – APHP, Paris<br>**Dr. Jessica Miroir.** Jean Perrin Centre, Clermont-Ferrand<br>**Corinne Millardet.** Jean Perrin Centre, Clermont-Ferrand

SUMMARY

I. RATIONALE OF THE STUDY AND SCIENTIFIC JUSTIFICATION 14

I.1. MANAGEMENT OF EARLY STAGE CANCERS OF THE ORAL CAVITY AND OROPHARYNX 14
I.2. POSTOPERATIVE RADIOTHERAPY FOR EARLY STAGE CANCERS OF THE ORAL CAVITY AND OROPHARYNX 14
I.3. HYPOFRACTIONATED STEREOTACTIC RADIOTHERAPY OF THE OPERATING BED IN EARLY STAGE CANCERS OF THE ORAL CAVITY AND OROPHARYNX WITH HIGH RISK MARGIN 17
I.4. STATEMENT OF ASSUMPTIONS AND OBJECTIVES 18
I.5. BENEFIT-RISK RATIO 18
I.6. EXPECTED BENEFITS 19

II. OBJECTIVES OF THE STUDY 19

II.1. MAIN OBJECTIVE 19
II.2. SECONDARY OBJECTIVES 19

III. STUDY DESCRIPTION 20

IV. RESEARCH DESIGN 20

IV.1. PRIMARY ENDPOINT 20
IV.2. SECONDARY ENDPOINTS 20

V. STUDY POPULATION 21

V.1. INCLUSION CRITERIA 21
V.2. NON-INCLUSION CRITERIA 21
V.3. DESCRIPTION OF STOP RULES 22

VI. RESEARCH METHODOLOGY 23

VI.1. DESCRIPTION OF THE RESEARCH METHODOLOGY 23
VI.2. RECRUITMENT PROCEDURES 24
VI.3. INCLUSION ASSESSMENT 24
VI.4. ASSESSMENT DURING STEREOTACTIC RADIOTHERAPY TREATMENT 25
VI.5. POST-TREATMENT FOLLOW-UP ASSESSMENT 25
VI.6. STUDY TIMELINE 29
VI.7. TOXICITY ASSESSMENT 29
VI.8. EFFECTIVENESS EVALUATION 29
VI.9. QUALITY OF LIFE ASSESSMENT 31

VII. BIOLOGICAL STUDY 32

VIII. TREATMENT UNDER STUDY: STEREOTACTIC RADIATION THERAPY 32

VIII.1. DESCRIPTION OF THE TREATMENT 32
VIII.2. DOSES, ADMINISTRATION MODALITIES AND DURATION OF TREATMENT 38

IX. STATISTICAL CONSIDERATIONS 41

IX.1. NUMBER OF TOPICS TO INCLUDE 41
IX.2. DATA ANALYSIS: GENERAL 41
IX.3. MAIN ANALYSIS 42
IX.4. SECONDARY ANALYSES 43
IX.5. METHOD OF ACCOUNTING FOR MISSING, UNUSED OR INVALID DATA 43
IX.6. RESPONSIBLE FOR ANALYSIS 44

X. INDEPENDENT OVERSIGHT COMMITTEE 44

XI. SECURITY ASSESSMENT – ADVERSE EVENT MANAGEMENT 44

XI.1. DEFINITIONS 44
XI.2. REPORTING OF SERIOUS ADVERSE EVENTS 47

XII. REPORT 50

XIII. RIGHT OF ACCESS TO SOURCE DATA AND DOCUMENTS 51

XIII.1. ACCESS TO DATA 51
XIII.2. SOURCE DATA 51
XIII.3. DATA PRIVACY 51

XIV. QUALITY CONTROL AND ASSURANCE 52

XIV.1. INVESTIGATOR AND SPONSOR ENGAGEMENT 52
XIV.2. QUALITY ASSURANCE 53
XIV.3. QUALITY CONTROL 53

XV. ETHICAL CONSIDERATIONS 53

XV.1. COMMITTEE FOR THE PROTECTION OF PERSONS 53
XV.2. PATIENT INFORMATION AND WRITTEN INFORMED CONSENT FORM 54
XV.3. AMENDMENTS TO THE PROTOCOL 54

XVI. DATA PROCESSING AND RETENTION OF DOCUMENTS AND DATA RELATING TO RESEARCH 54

XVI.1. DATA COLLECTION AND PROCESSING 54
XVI.2. CNIL 55
XVI.3. ARCHIVING 55

XVII. INSURANCE 55

XVIII. COMMUNICATION - PUBLICATION RULES 56

XIX. BIBLIOGRAPHY 57

XX. LIST OF APPENDICES 61

SYNOPSIS	
**PROMOTER**	**JEAN PERRIN CENTER**<br>58, rue Montalembert - BP 392 - 63011 CLERMONT-FERRAND
**PARTNER**	**GORTEC *(Head and Neck Radiotherapy Oncology Group)***<br>CHU Bretonneau – CORAD<br>2 Bd Tonnellé<br>37044 Tours cedex 9
**STUDY IDENTIFICATION**	**Sponsor Code**: GORTEC 2017-03<br>**RCB ID**: 2017-A02058-45
**VERSION & DATE**	**VERSION 2.0 OF November 25, 2019**
**TITLE OF THE STUDY**	Multicenter phase II study of postoperative hypofractionated stereotactic radiotherapy of early stage cancers of the oropharynx and oral cavity with high risk margins.
**SHORT TITLE**	STEREO – POSTOP *(Stereotactic – PostOperative)*
**COORDINATING INVESTIGATORS**	**Dr Julian BIAU, Principal Investigator**<br>Department of Radiotherapy - Jean Perrin Centre<br>58, rue Montalembert – 63011 Clermont-Ferrand<br>Phone : 04 73 27 81 42 / Fax : 04 73 27 81 25<br>@ Julian.biau@clermont.unicancer.fr<br><br>**Dr Michel LAPEYRE,** Co-Principal Investigator<br>Department of Radiotherapy<br>Centre Jean Perrin<br>58, rue Montalembert – 63011 Clermont-Ferrand<br>Phone : 04 73 27 81 42 / Fax : 04 73 27 81 25<br>@ Michel.lapeyre@clermont.unicancer.fr<br><br>**Prof. Jean BOURHIS,** ENT Intergroup - GORTEC<br>Head of the Radiation Oncology Department, CHUV<br>Hospital Building, Rue du Bugnon 46, CH-1011 Lausanne, Switzerland<br>Tel: +41 21 314 46 65 - Fax: +41 (0)21 314 46 01<br>@ Jean.bourhis@chuv.ch
**INDICATION**	Squamous cell carcinoma of the oral cavity (excluding the lips) or oropharynx operated with risk margins
**STUDY DESIGN**	Phase II, multicenter, national, non-randomized, open-label trial
**NUMBER OF PLANNED CENTRES**	20 – 25 centres
**NUMBER OF PATIENTS**	90 patients

OBJECTIVES OF THE STUDY

Main Objective

To assess the late toxicity of hypofractionated stereotactic radiotherapy of the operating bed of early stage cancers of the oral cavity operated on with high risk margins.

Secondary Objectives

- Assessing local and regional control
- Assessing acute toxicity
- To assess recurrence-free survival and overall survival
- Assessing patients' quality of life
- Assessing the nutritional impact
- Determining Predictors of Toxicity

Exploratory objective:

- to study the dosimetric impact of the addition of non-coplanar arcs to the Novalis accelerator-modulated volumetric arc therapy technique, and to study the dose-toxicity relationship on the first 10 patients treated with this technique
-

INCLUSION CRITERIA

1. Squamous cell carcinoma of the oral cavity (excluding the lips) or oropharynx operated on
2. pT1 or pT2 (UICC 7th edition 2009)
3. Indication for irradiation of the operating bed (retained in RCP) with at least one of the following criteria:
   - ❖ *Positive margin R1 (without retained surgical revision)*
   - ❖ *Margin< 5mm (without surgical revision retained)*
   - ❖ *Estimated margin at risk, with uncertain histopathological margin (without retained surgical revision)*
4. N0 after surgical exploration of the cervical lymph node areas (dissection or sentinel lymph node) or pN1 without capsular rupture (carcinologic dissection)
5. Age> 18 years old
6. ECOG Status ≤ 2
7. Signing of informed consent before any specific protocol procedure
8. Affiliation to or beneficiary of a social security scheme

CRITERIA FOR NON-INCLUSION

1. Other histology than squamous cell carcinoma
2. pT3 or pT4
3. pT2 > 3cm and R1 with concomitant chemotherapy decided in RCP
4. Presence of lymphatic emboli requiring cervical lymph node irradiation
5. Indication for cervical lymph node irradiation decided by RCP

6. Missing at least one of the following:
   - *Preoperative imaging: CT scan or MRI*
   - *Endoscopy report*
   - *Operative report*
   - *Histopathological report*
7. Previous radiotherapy treatment on the cervicofacial region
8. Distant metastasis(s)
9. Pregnant or breastfeeding woman
10. Patient (male or female) of childbearing potential not taking adequate contraceptive measures
11. Concurrent participation in another interventional therapeutic trial within 4 weeks prior to inclusion
12. Other associated or previous cancer within 5 years prior to inclusion except in situ cervical cancer or controlled basal cell carcinoma
13. Persons deprived of their liberty, under guardianship or curatorship, or who are unable to undergo the medical follow-up of the trial for geographical, social or psychological reasons.

PROCESSING METHODS

Stereotactic radiotherapy 36 Gy in 6 fractions:

1. Restraint: Stereotactic Custom Restraint Mask

2. Volumes:

- CTV = Initial tumor bed including the positive or close margin + 5-10 mm. If flap, also include the normal tissue/flap junction + 5 mm flap next to the tumor bed. Peritumoral sutures in relation to mucosal healthy tissue will be included.
- PTV = CTV + 2 mm (to be adapted according to the practice of the centre)

3. Doses

- Accelerators dedicated to stereotactic and equipped multi-purpose accelerators are permitted.
- The prescription is made to encompass at least 95% of the PTV in the prescribed physical dose, which is 36Gy. The prescription will be RCMI or Cyberknife type.
  - In an RCMI type prescription, this prescribed physical dose of 36Gy will correspond to the 100% isodose. It is recommended that D5% ≤ 39.6 Gy (110% of the prescribed dose of 36Gy). The coverage of the PTV will be as homogeneous as possible due to the post-operative situation.
  - In Cyberknife prescription, the prescription isodose of 36Gy will be determined to limit heterogeneities within the target volume as much as possible (classically 80% isodose). It is recommended that D20% ≤ 39.6 Gy (110% of the prescribed dose of 36Gy).

4. Spreading: 11-13 days, at the rate of 3 fractions per week. A minimum of 36 hours will be required between 2 fractions.

Study Outline

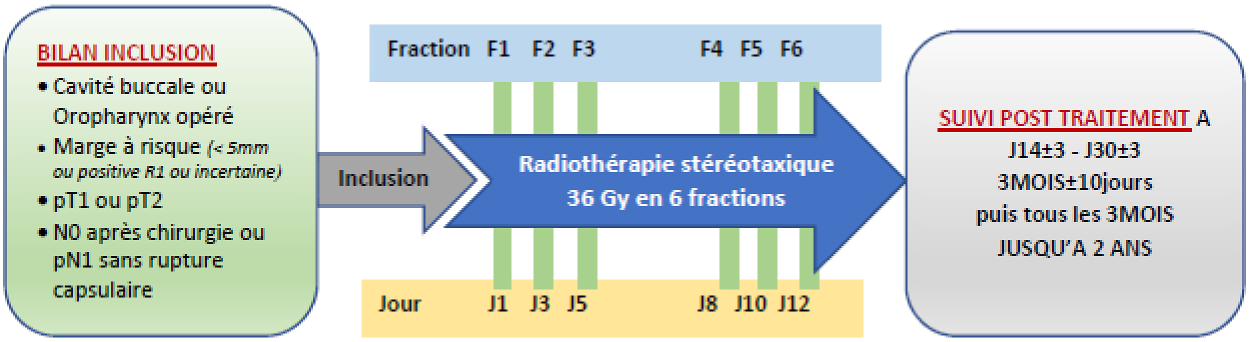

EVALUATION CRITERIA

Primary endpoint

Rate of patients with late toxicity at 2 years grade ≥ 3 according to the NCI - CTCAE V4.03 classification, in connection with hypofractionated stereotactic radiotherapy.

Secondary endpoints

- **Local control at 2 years**. Will be considered as an event: local recurrence (T) documented in the area of the operating bed. The diagnosis of local recurrence requires histological confirmation.
- **Locoregional control at 2 years**. The following are considered to be events: local recurrence (T) and/or lymph node recurrence (N), i.e. ipsilateral or contralateral cervical positive lymphadenopathy(es). The diagnosis of regional recurrence requires confirmation by imaging and/or histologic.
- **Grade ≥ 3** (≤ 3 months) acute toxicity in connection with hypofractionated stereotactic radiotherapy, according to NCI CTCAE V4.03 classification
- **2-year recurrence-free survival (RSS):** defined as the time interval between the date of inclusion and the date of the first cancer event (local recurrence, regional recurrence, distant metastasis) or death from any cause.
- **2-year overall survival (OS):** defined as the time interval between the date of inclusion and the date of death from any cause.
- **Quality of Life (QoL):** according to the EORTC QLQ-C30 and EORTC-H&N35 questionnaires

- **Nutritional impact**, assessed by patient weight and feeding tube use.
- **Predictive factors of toxicity at 2 years** : clinical and/or dosimetric factors associated with late severe toxicity

STATISTICAL CONSIDERATIONS

Estimating the number of patients needed

With a one-step Fleming plan, the calculation of the number of patients needed takes into account:

- The primary endpoint: severe toxicity at 2 years limited to 5-15%, minimum N is equal to 67 patients (unilateral, $\alpha = 0.05$, $\beta=0.10$)
- and the secondary endpoint: 2-year local control of 80-90%, minimum N is equal to 83 patients (unilateral, $\alpha = 0.05$, $\beta=0.20$)

In order to compensate for possible loss to follow-up, 90 patients will be included.

Method of statistical analysis

All statistical analyses will be performed with the *Stata software* (version 13, StataCorp, College Station, USA) in accordance with the recommendations of the *International Conference on Harmonization-Good Clinical Practice*. An analysis plan will be drawn up before the database is frozen.

QUALITY CONTROL OF RADIOTHERAPY

Dummy run

The evaluation of the quality of the radiotherapy will be carried out by the Quality Assurance team of GORTEC.

All the centres will carry out a test case (*Dummy run*). This 2-step procedure will contain **i) delineation and ii) dosimetry** according to the protocol recommendations of a test case that will be provided. This test case will be reviewed centrally by the GORTEC Quality Assurance team. The participation of the centers will be confirmed or rediscussed, in particular depending on the compliance results of *Dummy run.*

Retrospective control

A Quality Assurance control will also be carried out retrospectively on all patients treated. Each center will have to upload its patients' files to the dedicated platform within 30 days of the end of stereotactic radiotherapy.

PROVISIONAL SCHEDULE OF THE STUDY

**Duration of inclusions**	48 months
**Maximum processing time**	11 – 13 days
**Post-treatment follow-up time**	24 months

**Overall duration of the study**	72 months
**3rd quarter 2017**	Submission CPP and ANSM
**Q1 2018**	Initiation of the centers + Inclusion of the first patient
**Q1 2022**	Inclusion of the last patient
**Q1 2024**	Last follow-up visit
**Q3 2024**	Analysis and end-of-study report

124

LIST OF ABBREVIATIONS

2D: 2 Dimensions (Two-Dimensional)
3D: 3 Dimensions (three-dimensional)
BED: Effective Biological Dose
CIS: Carcinoma In Situ
CL: ControLateral
cLDA: constrained Longitudinal Data Analysis
CTV: Microscopic Target Volume
FDG: Fluoro-DeoxyGlucose
GORTEC: Head and Neck Radiotherapy Oncology Group
Gy: Gray
HL: HomoLateral
HPV: Human Papilloma Virus
95% CI: 95% Confidence Intervals
ICRU: *International Commission on Radiation Units and measurements*
IDSMC: Independent Trial Monitoring Committee
IGRT: image-guided radiotherapy
MRI: Magnetic Resonance Imaging
^{192}Ir: Irridium-192
kV: kilo-Volt
LDR: Low Dose Rate Brachytherapy
MV: Mega Volt
SRO: Organ at Risk
PDR: pulsed flow brachytherapy
PRV: SRO Forecast Volumes
PTV: Projected target volume
QLQ-C30 Quality of Life Questionnaire C30 (Questionnaire de Qualité de vie C30)
QLQ-HN35: Quality of Life Questionnaire Head and Neck 35
IMRT: Intensity-modulated conformal radiotherapy
RCP: Multidisciplinary Consultation Meeting
RT: Radiotherapy
SFORL: French Society of Otorhinolaryngology and Face and Neck Surgery
OS: Overall Survival
SRS: Recurrence-Free Survival
PET: Positron Emission Tomography
CT scan: Computed tomography
Tk: cell repopulation coefficient
TP: tumor doubling time
TPS: *Treatment Planning System*

I. RATIONALE OF THE STUDY AND SCIENTIFIC JUSTIFICATION

I.1. Management of early stage cancers of the oral cavity and oropharynx

Early stage cancers of the oral cavity and oropharynx are in the majority of cases squamous cell carcinomas. The main risk factors are tobacco, alcohol and HPV infection. Their impact is growing (1). Their care is multidisciplinary. In localized forms, surgery is generally the first-line treatment when it is possible (2). Surgical exploration of the cervical lymph nodes is most often performed either by dissection or sentinel lymph node (3–5). Negative resection margins (>5 mm) are recommended (6,7). In case of positive margins, new surgery should be proposed when possible. When this is not retained, post-operative radiotherapy is indicated (8–11). This post-operative radiotherapy may be limited to the tumour bed in the case of pT1-2 and negative cervical exploration (or if only one lymph node is affected without capsular rupture in the case of a carcinological dissection) (11,12). Two types of radiotherapy are used when irradiation of the operating bed alone is indicated: brachytherapy or normofractionated external beam radiotherapy (recommended in intensity-modulated conformal radiotherapy [IMRT]).

I.2. Postoperative radiotherapy for early stage cancers of the oral cavity and oropharynx

I.2.1 Postoperative brachytherapy

Brachytherapy is a highly conformal technique that allows a high dose to be delivered, in a small volume, in a short time (13–15). However, it is not always feasible: involvement of the gum or retromolar trigon, a distance of less than 5 mm from the gum, infiltration of the intermaxillary commissure, or extension to the parapharynx, to the cavum for an oropharyngeal tumor, to the union of the three folds or to the larynx. In addition, brachytherapy requires highly trained teams, hospitalization of the patient, general anesthesia, and adapted infrastructure.

Goineau et al. (16) reported the results of 112 patients treated with postoperative low-dose-rate (LDR) interstitial brachytherapy by ^{192}Ir for squamous cell carcinomas of the mobile tongue. The local control rate at 2 years was 79% and at 5 years was 76%. The overall survival rate at 2 years was 72% and at 5 years was 56%. 22% of patients experienced radionecrosis requiring surgery. 8% of patients presented with chronic pain requiring the use of morphine. These results were similar to those of other smaller retrospective studies (13,17,18). In the study by Lapeyre et al. (18) the local control rate was 81% and the 5-year overall survival rate was 70% for patients with T1/T2-N0 tumors. Strnad

et al. (19) reported the results of the largest brachytherapy study with 385 patients. Patients were treated with pulsed flow brachytherapy (PDR). Brachytherapy was postoperative in 85% of cases. The local control and overall 5-year survival rates were 85% and 69% respectively. The rates of severe late toxicity of mucosal ulceration and osteoradionecrosis were 10 and 5%, respectively.

I.2.2 Postoperative intensity-modulated radiotherapy (IMRT)

Postoperative normofractional IMRT is, along with brachytherapy, the second option of irradiation but mobilizes the patient for 6 to 7 weeks (20–24). No randomised trials compared the carcinologic outcomes and toxicity profiles of postoperative brachytherapy with postoperative IMRT in localised cancers of the oropharynx and oral cavity. Studies reporting the results of postoperative normofractional IMRT in cancers of the oropahrynx and oral cavity often group together localized and locally advanced cancers. Collan et al. (23) reported a grade ≥ 3 acute mucositis rate of 25% in 102 patients. For late toxicities, 5% of patients required a permanent feeding tube and 30% had a grade 2 xerostomia. Geretschläger et al. (22) reported the results of 53 patients treated for oral cancers. They reported a rate of grade 3 acute mucositis of 36%, grade 2 late xerostomia of 6%, osteoradionecrosis of 4%, grade 3 mucosal ulceration of 2% and 9% permanent feeding tube. Other studies have reported similar toxicity profiles (20,21,24). Overall, rates of severe acute mucositis and permanent feeding tube were reported in 11-36% and 5-10% respectively. Rates of severe late mucosal ulceration (grades 3-4) ranged from 2 to 4% and osteoradionecrosis from 0 to 5% (20–24).

I.1.3 Hypofractionated stereotactic radiotherapy in head and neck cancers

Hypofractionated stereotactic radiotherapy delivers an ablative dose of radiation to extracranial tumors in one or more fractions using advanced planning and treatment techniques (25–28). This technique has the advantage of offering a highly conformational treatment, in a limited number of fractions, with a strong dose gradient leading to limited irradiation of the surrounding healthy tissues (28). The use of this technique is currently possible due to the rapid expansion of the French stereotactic technical park, but needs to be well supervised (27,29–35). Stereotactic radiotherapy in head and neck cancers has mainly been studied in cases of re-irradiation or boost.

- ***Stereotactic radiotherapy in re-irradiation***

Stereotactic radiotherapy studies in re-irradiation of head and neck cancers give us information about the acute toxicity of this technique. Information on late toxicity and efficacy would be difficult to compare.

Heron et al. (31) reported the results of a Phase 1 dose-escalation study of stereotactic radiotherapy in case of re-irradiation for recurrences of squamous cell carcinomas of the head and neck. A total of 25 patients received doses ranging from 5Gy to 8.8Gy per fraction, in 5 fractions over 2 weeks. No Grade ≥ 3 limiting dose toxicity has been reported. The same team reported its results on 96 patients (33), showed a dose-response effect with a higher local control rate for tumors receiving higher doses. An update of these results has been published (35). This study included 132 patients treated with a median dose of 44Gy (35-50Gy). They found a higher incidence of acute (but not late) toxicity for patients with a tumor volume of more than 25 cm3.

Two non-randomized phase II studies reported the results of stereotactic radiotherapy with concomitant cetuximab in the setting of recurrence or second cancer in the irradiated territory, which were not operable. The first study by Lartigau et al. (29) included 56 patients treated with a dose of 36 Gy in 6 fractions over 2 weeks on Cyberknife. In terms of acute toxicity, grade ≥ 3 mucositis was found in 4/56 patients and grade ≥ 3 dysphagia in 3/56 patients. The second study by Vargo et al. (36) included 48 patients treated with a dose of 40-44 Gy in 5 fractions over 1-2 weeks with different types of accelerators (Cyberknife, Trilogy or Truebeam). 3/48 patients experienced at least one type of grade 3 acute toxicity: 1 patient had mucositis and epidermatitis, 1 patient had dysphagia and the third had epidermatitis. There was no significant difference in terms of carcinologic results, toxicity or feasibility between the different types of accelerators. In these two studies by Lartigau et al.(29) and Vargo et al.(36), the authors concluded that this treatment was feasible with acceptable toxicities.

Another study by Vargo et al.(34) studied 28 patients with margin at risk treated postoperatively with stereotactic radiotherapy in irradiated territory at a dose of 40 Gy in 5 fractions. There was 39% oral and oropharyngeal cavity in this study. No grade 3 acute toxicity was observed. In another study by Vargo et al. (30) of 12 inoperable elderly patients with stage III-IV tumors in the non-irradiated territory, treated with stereotactic radiotherapy alone (44 Gy in 12 fractions), grade 3 acute toxicity was observed in 1/12 dysphagia patients and grade 3 late toxicity was observed in 1/12 patients also with mucositis type.

- ***Stereotactic radiotherapy in boost***

Hypofractionated stereotactic radiotherapy in head and neck cancers has also been evaluated as a boost after initial normofractionated IMRT. Al-Mamgani et al. (37) studied stereotactic radiotherapy as a therapeutic option for boosting oropharyngeal cancers not eligible for brachytherapy. 51 patients received a 3 x 5.5Gy boost after an IMRT of 46Gy on the primary tumor and neck. The authors reported that overall tolerability was good, there were no discontinuations of treatment for toxicity, and no grade 4 or 5 acute or late toxicities. The cumulative incidence of Grade ≥ 2 late toxicity was

28%. Among the recurrence-free patients at 2 years (n=20), there was only 1 patient with a feeding tube and 2 patients with grade 3 xerostomia. This same team reported its efficacy and toxicity results of two different boost modalities for T1-T2 squamous cell carcinomas of the oropharynx: hypofractionated stereotactic radiotherapy or brachytherapy (38). The 3-year local control rates for stereotactic radiotherapy were 97% and 94% respectively (p=0.33). The 3-year recurrence-free survival rates were 92% and 86% (p=0.15) respectively and the overall survival rates were 81% and 83% (p=0.82). The feeding tube rates were 17% and 20% respectively (p=0.47). The rates of late dysphagia grade ≥ 2 were 11% and 8% (p=0.34) and xerostomia of 16% and 12% (p=0.28), respectively. The authors concluded that due to the heavy logistics required for implantation, the need for a trained team and the risks of general anesthesia and perioperative complications, stereotactic radiotherapy seemed to be the optimal option for boosting localized oropharyngeal cancers compared to brachytherapy.

I.3. Hypofractionated stereotactic radiotherapy of the operating bed in early stage cancers of the oral cavity and oropharynx with high risk margin

In our study, we hypothesize that the safety and efficacy profile of stereotactic radiotherapy of the operative bed in localized cancers of the oral cavity and oropharynx with risk margins will be similar to that of other radiotherapy techniques: brachytherapy or normofractionated IMRT. Stereotactic radiotherapy could become the third therapeutic option in this indication. The use of stereotactic radiotherapy of the operating room for localized cancers of the oropharynx and oral cavity with risk margins would have several advantages:

- Highly conformal technique such as brachytherapy
- Fewer contraindications than brachytherapy due to tumor extension and/or implantation difficulties
- Carrying out the treatment on an outpatient basis, without hospitalization or general anesthesia, unlike brachytherapy
- Short spreading: 6 fractions over 2 weeks vs. 30-33 fractions over 6-7 weeks for fractional IMRT.

The post-operative use of hypofractionated radiotherapy (excluding re-irradiation) has already been reported in ENT cancers, particularly in the context of mucosal melanomas (39–41). Most of these studies did not use stereotactic irradiation. Wu et al. (41) reported in 2010 their experience on 27 patients, 10 of whom were treated at a dose of 5x6Gy over 2 weeks and 8 patients at a dose of

3x7-8Gy over 3 weeks. In this study, 3/27 patients experienced Grade 3 acute toxicity (2 epidermatitis and 1 mucositis) and no patients had Grade 4 acute toxicity. With a median follow-up of almost 4 years, no grade ≥ 3 toxicity was reported.

In total, a dose of 36Gy in 6 fractions over 2 weeks seems to be a good compromise postoperatively in terms of benefit/risk. To determine the effective biological doses (BEDs), we used the following formula to take into account the tumor doubling time (Tp), the spread of radiotherapy and the cell repopulation coefficient (Tk) (42,43):

$$\mathrm{BED} = nd\left(1 + \frac{d}{\alpha/\beta}\right) - \frac{Ln2(T - T_k)}{\alpha T_p}$$

With this model, we obtain for the tumor bed a BED10 of 64.2 Gy for a Tk at 21 days (equivalent to the BED_{10} of 60 Gy in 30 fractions); a BED10 of 54.4 Gy for acute side effects (equivalent to the BED10 of 74 Gy in 37 fractions); a BED3 of 108Gy for late side effects (equivalent to the BED3 of 66 Gy in 33 fractions).

I.4. Statement of Assumptions and Objectives

In this study, we hypothesize that the safety and efficacy profile of stereotactic radiotherapy of the operative bed in localized cancers of the oral cavity and oropharynx with risk margins will be similar to that of other radiotherapy techniques: brachytherapy or normofractionated IMRT. Stereotactic radiotherapy could become the third therapeutic option to treat localized cancers of the oral cavity and oropharynx with risk margin.

The main objective of the study will be to evaluate the late toxicity of stereotactic radiotherapy of the operating bed of localized cancers of the oral cavity operated with risk margins.

I.5. Benefit-risk ratio

The expected benefits for the patient are:

- Shorter treatment than normofractionated IMRT: 2 weeks vs. 6-7 weeks
- Treatment less severe than brachytherapy: no hospitalization or general anesthesia
- Maintaining a good quality of life during treatment
- Efficacy and tolerability profiles at least equivalent to standard treatments
- Good accessibility to innovative treatment throughout the country

The expected benefits in terms of public health are:

- Prospective evaluation of an innovative treatment

- Standardization and optimization of stereotactic radiotherapy in head and neck cancers (supported by GORTEC's high-level quality control) leading to improved professional practices
- Development of an innovative treatment with an expected cost-effectiveness ratio

The foreseeable and known risks are the same as for standard treatments in this indication, which are brachytherapy and normofractional IMRT.

The evaluation of acute and late toxicities is one of the secondary objectives of the study. The toxicity profile will be assessed using the NCI-CTCAE v.4.03 scale. Any toxicity, and its potential attributable to stereotactic radiation therapy, will be graded and recorded by the investigator. Particular attention will be paid to the evaluation of the following toxicities possibly related to stereotaxy: mucositis, dysphagia, xerostomia, mandibular osteonecrosis, soft tissue necrosis, epidermatitis and fibrosis.

I.6. Expected benefits

In the event that this study confirms that the safety and efficacy profile of stereotactic radiotherapy of the operating bed in localized cancers of the oral cavity and oropharynx with risk margins is similar to that of other radiotherapy techniques (brachytherapy or IMRT), stereotactic radiotherapy could become the third therapeutic option to treat localized cancers of the oral cavity and oropharynx with margin at risk.

II. OBJECTIVES OF THE STUDY

II.1. Main objective

The main objective is to evaluate the late toxicity of stereotactic radiotherapy of the operating bed of localized cancers of the oral cavity operated with risk margins.

II.2. Secondary Objectives

The secondary objectives will be to evaluate at 2 years:

- The local control rate
- The locoregional control rate
- Recurrence-free survival
- Overall survival
- Patients' quality of life
- Nutritional impact

- Acute toxicity (grade ≥ 3 occurring during treatment and up to 3 months after the end of treatment)
- Determining Predictors of Toxicity

Exploratory objective:

- to study the dosimetric impact of the addition of non-coplanar arcs to the Novalis accelerator-modulated volumetric arc therapy technique, and to study the dose-toxicity relationship on the first 10 patients treated with this technique

III. STUDY DESCRIPTION

This is a Category I, non-drug, Phase II, multicentre, national, non-randomized, open-label human research with the primary outcome of which is the rate of severe late toxicity (grade ≥ 3).

IV. RESEARCH DESIGN

IV.1. Primary endpoint

Rate of patients with late toxicity at 2 years grade ≥ 3 according to the NCI - CTCAE V4.03 classification in connection with hypofractionated stereotactic radiotherapy., i.e. grade ≥ 3 toxicities occurring beyond 3 months after the end of stereotactic radiotherapy.

IV.2. Secondary endpoints

- **Local control at 2 years**. Will be considered as an event: local recurrence (T) documented in the area of the operating bed. The diagnosis of local recurrence requires histological confirmation.
- **Locoregional control at 2 years**. The following are considered to be events: local recurrence (T) and/or lymph node recurrence (N), i.e. ipsilateral or contralateral cervical positive lymphadenopathy(es). The diagnosis of regional recurrence requires confirmation by imaging and/or histologic.
- **Grade ≥ 3 acute toxicity** (toxicities occurring during treatment up to ≤ 3 months after the end of treatment) in connection with hypofractionated stereotactic radiotherapy, according to the NCI classification - CTCAE V4.03
- **2-year recurrence-free survival (RSS):** defined as the time interval between the date of inclusion and the date of the first cancer event (local recurrence, regional recurrence, distant metastasis) or death from any cause.

- **2-year overall survival (OS):** defined as the time interval between the date of inclusion and the date of death from any cause.
- **Quality of Life (QoL):** according to the EORTC QLQ-C30 and EORTC-H&N35 questionnaires
- **Nutritional impact**, assessed by patient weight and feeding tube use.
- **Predictive factors of toxicity at 2 years** : clinical and/or dosimetric factors associated with late severe toxicity.

V. STUDY POPULATION

V.1. Inclusion criteria

1. Squamous cell carcinoma of the oral cavity (excluding the lips) or oropharynx operated on
2. pT1 or pT2 (UICC 7th edition 2009)
3. Indication for irradiation of the operating bed (retained in RCP) with at least one of the following criteria:
    - *Positive margin R1 (without retained surgical revision)*
    - *Margin< 5mm (without surgical revision retained)*
    - *Estimated margin at risk, with uncertain histopathological margin (without retained surgical revision)*
4. N0 after surgical exploration of the cervical lymph node areas (dissection or sentinel lymph node) or pN1 without capsular rupture (carcinologic dissection)
5. Age> 18 years old
6. ECOG Status ≤ 2
7. Signing of informed consent before any specific protocol procedure
8. Affiliation to or beneficiary of a social security scheme

V.2. Non-inclusion criteria

1. Other histology than squamous cell carcinoma
2. pT3 or pT4
3. pT2 > 3cm and R1 with concomitant chemotherapy decided in RCP
4. Presence of lymphatic emboli requiring cervical lymph node irradiation
5. Indication for cervical lymph node irradiation decided by RCP
6. Missing at least one of the following:
    - *Preoperative imaging: CT scan or MRI*
    - *Endoscopy report*

- *Operative report*
- *Histopathological report.*

7. Previous radiotherapy treatment on the cervicofacial region
8. Distant metastasis(s)
9. Pregnant or breastfeeding woman
10. Patient (male or female) of childbearing potential not taking adequate contraceptive measures
11. Concurrent participation in another interventional therapeutic trial within 4 weeks prior to inclusion
12. Other associated or previous cancer within 5 years prior to inclusion except in situ cervical cancer or controlled basal cell carcinoma
13. Persons deprived of their liberty, under guardianship or curatorship, or who are unable to undergo the medical follow-up of the trial for geographical, social or psychological reasons.

V.3. Description of stop rules

V.3.1 Permanent or temporary cessation of a person's participation

All patients included in this study will have the right to leave the study at any time, without having to justify the reason and without this calling into question their usual medical follow-up.
The reasons for leaving the study must be related to the following criteria:

- Withdrawal of consent: the patient will be free to stop the study at any time and without prejudice to his or her further treatment. The patient will have to specify whether he or she only wishes to stop the current treatment, or whether he or she also wishes to stop the monitoring provided for in the protocol or whether he or she wishes to have the data already collected in the database about him or her destroyed.
- Unacceptable toxicity attributed to treatment
- Pregnancy
- Non-compliance with the protocol
- Patient lost to follow-up
- Death.

V.3.2 Premature discontinuation of study treatment

Treatment may be discontinued prematurely for the following reasons:

- Toxicity
- Disease progression
- Refusal to continue the trial, withdrawal of consent

- Major breach of protocol.

Whenever possible, patients who have discontinued treatment prematurely will be monitored according to the protocol.

V.3.3 Discontinuation of the study

The end of the study is expected when the last patient has completed their follow-up at 2 years. The study may be stopped prematurely if the toxicity is too high or the inclusion rate is too low. The independent oversight committee will make its recommendations. It is the developer who will decide on the stoppage.

V.3.4 Period of deferral and participation in other research

Prohibition from participating in any other interventional study during participation in the study, i.e. for 2 years after inclusion (and in the 4 weeks prior to inclusion).

VI. RESEARCH METHODOLOGY

VI.1. Description of the research methodology

This is a multicenter, national, non-randomized, phase 2, open-label trial that aims to evaluate the toxicity and efficacy profile of hypofractionated stereotactic radiotherapy of the operative bed of localized cancers of the oral cavity and oropharynx with risk margins. The experimental design was chosen according to a Fleming design at one stage without intermediate analysis because of the primary endpoint which will be evaluated at 2 years (severe late toxicity).

Figure 1: Test diagram

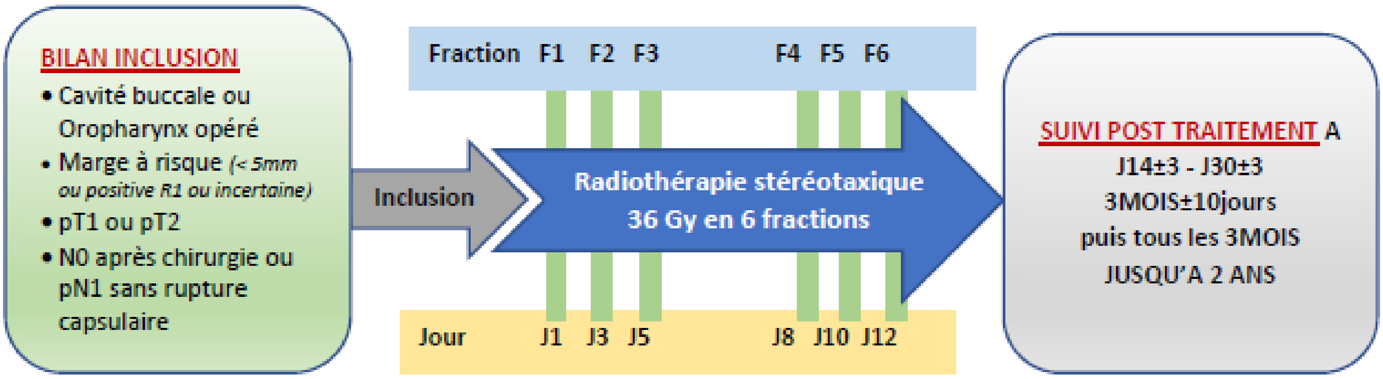

VI.2. Recruitment procedures

Patients will be screened in a Multidisciplinary Consultation Meeting (RCP) and those who are potentially eligible for the study (indication of irradiation of the operating bed) will be seen in consultation by a radiotherapist and/or a surgeon, who will offer them to participate in the trial. Patients who meet the inclusion criteria without non-inclusion criteria, and who have signed informed consent will be eligible to participate in this trial.

Inclusion is done via the electronic CRF and must be performed before the start of stereotactic radiotherapy.

The included patients will be treated according to the modalities provided for in the trial.

VI.3. Inclusion assessment

Patients eligible for the trial who have signed their consent to participate will be required to have an **initial work-up within 28 days prior to the start of stereotactic radiotherapy.**

This assessment includes:

- Collection of socio-demographic data
- Collection of major medical and/or surgical history
- Collection of concomitant treatments
- History of ENT cancer (date of diagnosis, location, cTNM, histology, HPV status, tumour surgery data, etc.)
- A sample of the tumour taken during diagnosis or surgery will be sent to the sponsor. The analysis of the HPV (immunohistochemistry p16) status of the tumor will be performed in case it was not done by the center initially.
- Clinical examination including usual weight, day weight and height
- Assessment of general condition according to WHO status *(Annex 2)*
- Smoking status (number of packs/year)
- Alcohol consumption
- Type of feed (solid, liquid, mixed, etc.)
- Tumor bed examination (clinical +/- nasofibroscopy)
- Examination of the mouth, throat and neck. Dental work should be done before treatment begins.
- Pregnancy test in women of childbearing potential: urine or blood test within 14 days (maximum) before starting stereotactic radiotherapy
- Chest CT scan: should be performed only if the time between the date of the initial CT scan and the start date of stereotactic radiotherapy is more than 3 months.

- EORTC QLQ-C30 and H&N35 questionnaire: To be completed by the patient after inclusion and before starting stereotactic radiotherapy.

Before a patient is included, you will need to ensure that the following documents/imaging are available:

- *Pre-operative imaging: CT scan or cervicofacial MRI*
- *The endoscopy report*
- *The operative report*
- *The anatomical and pathological report*

IMPORTANT REMINDER

The time between the date of surgery of the primary tumor and the date of the first fraction of stereotactic radiotherapy should be less than or equal to 6 weeks (maximum 8 weeks).

VI.4. Assessment during stereotactic radiotherapy treatment

The assessments will be carried out **on the day of the 4th fraction (expected day D8) and the day of the last fraction (expected day D12).**

They include:

- Clinical examination and weight
- Assessment of general condition according to WHO status
- Type of feed (solid, liquid, mixed, etc.)
- Acute toxicity assessments according to NCI-CTCAE v.4.03
- Collection of concomitant treatments

VI.5. Post-treatment follow-up assessment

Patients will be followed according to the recommendations of the *French Society of Otorhinolaryngology (SFORL) and Face and Neck Surgery* (46).

VI.5.1 Assessment during immediate follow-up visits

Follow-up visit at 14±3 days after the last stereotactic radiation therapy session

- Clinical examination including weight
- Assessment of general condition according to WHO status
- Tumor bed examination (clinical +/- nasofibroscopy)
- Examination of the mouth, throat and neck

- Type of feed (Solid, liquid, mixed...)
- Acute toxicity assessments according to NCI-CTCAE v.4.03
- Collection of concomitant treatments

__Follow-up visit at 30±5 days after the last stereotactic radiotherapy session__

- Clinical examination including weight
- Assessment of general condition according to WHO status
- Tumor bed examination (clinical +/- nasofibroscopy)
- Examination of the mouth, throat and neck
- Type of feed (solid, liquid, mixed, etc.)
- Acute toxicity assessments according to NCI-CTCAE v.4.03
- Collection of concomitant treatments
- EORTC QLQ-C30 and H&N35 Questionnaire

__Follow-up at 3 months ±10 days after the last stereotactic radiation therapy session__

- Clinical examination including weight
- Assessment of general condition according to WHO status
- Tumor bed examination (clinical +/- nasofibroscopy)
- Examination of the mouth, throat and neck
- Type of feed (solid, liquid, mixed, etc.)
- Acute toxicity assessments according to NCI-CTCAE v.4.03
- Carcinologic evaluation:
    - ❖ CT scan or cervicofacial MRI *(The choice between MRI and CT is left to the discretion of the investigator but follow-ups must be carried out with the same technique)*
    - ❖ Chest CT scan *(The use of PET-FDG is left to the choice of the investigators in each center if it is medically indicated)*

VI.5.2 Assessment during long-term monitoring visits

These visits will take place every **__3 months±10 days for up to 2 years__** after stereotactic radiotherapy and include:

- Clinical examination including weight
- Assessment of general condition according to WHO status
- Tumor bed examination (clinical +/- nasofibroscopy)

- Examination of the mouth, throat and neck
- Type of feed (solid, liquid, mixed, etc.)
- Late toxicity assessments according to NCI-CTCAE v.4.03
- Carcinologic assessment at **12 months and 24 months**
    - CT scan or cervicofacial MRI *(The choice between MRI and CT is left to the discretion of the investigator but follow-ups must be carried out with the same technique)*
    - Chest CT scan *(The use of PET-FDG is left to the choice of the investigators in each center if it is medically indicated)*
- EORTC QLQ-C30 and H&N35 questionnaire to be completed at **12 months and 24 months,** in the absence of local, locoregional or distance progression.

VI.5.3 Follow-up on recidivism

In the event of local, regional, locoregional or distant recurrence, patients will be monitored only for their vital status.

Data from salvage treatments will be collected in the CRF.

Table 1: Flow chart

PERIODE DE L'ESSAI	BILAN A L'INCLUSION	SUIVI PENDANT LA RADIOTHERAPIE		VISITES DE SUIVI POST RADIOTHERAPIE STEREOTAXIQUE									
**Visites n°**	**V0**	**V1**	**V2**	**V3**	**V4**	**V5**	**V6**	**V7**	**V8**	**V9**	**V10**	**V11**	**V12**
Semaines *(par rapport au debut de la radiothérapie)*	**S-4 à S0**	**S2**		**14 ± 3 jours**	**30±5 jours**	**3 mois** ±10 jours	**6 mois** ± 10 jours	**9mois** ±10 jours	**12 mois** ±10 jours	**15 mois** ±10 jours	**18 mois** ±10 jours	**21 mois** ±10 jours	**24 mois** ±10 jours
Jours *(par rapport au debut de la radiothérapie)*	***J-28 à J-1***	***J8***	***J12±1***										
Consentement éclairé signé	X												
Données socio-démographiques	X												
Antécédents médicaux / pathologies concomitantes	X												
Histoire de la maladie	X												
Statut tumoral HPV	X												
Test de grossesse *(sang ou urine)* *	X												
Critères d'inclusion / non inclusion	X												
Bilan clinique, poids, taille	X	X	X	X	X	X	X	X	X	X	X	X	X
Examen dentaire et soins adaptés	X												
Evaluation de l'état général (Classification OMS)	X	X	X	X	X	X	X	X	X	X	X	X	X
Examen ORL *(bouche, gorge et cou)*	X			X	X	X	X	X	X	X	X	X	X
Examen du lit tumoral *(clinique +/- nasofibroscopie)*	X			X	X	X	X	X	X	X	X	X	X
Toxicités (CTCAE v.4,03)		***Recueil des toxicités aigues***					***Recueil des toxicités tardives***						
Traitements concomitants	***Recueil des traitements concomitants***												
Compte-rendu d'endoscopie	X												
Compte-rendu de chirurgie	X												
Compte-rendu anatomo-pathologique	X												
Scanner ou IRM cervico-facial						X			X				X
Scanner thoracique*	(X)					X			X				X
PET-FDG						si besoin			si besoin				si besoin
Questionnaires Qdv EORTC QLQC30 et HN35	X				X				X				X
Bloc tumoral ***(fixé et inclus en paraffine)***	X												

Test de grossesse doit être réalisé dans les 14 jours maximum avant le début de la radiothérapie stéréotaxique
Scanner thoracique: A refaire à l'inclusion uniquement si le délai entre le scanner thoracique préopératoire et la date de début de la radiothérapie est supérieur à 3 mois.
En cas de récidive locale, régionale, locorégionale et/ou à distance, les patients seront suivis uniquement pour le statut vital

VI.6. Study timeline

Duration of inclusions 48 months
Maximum processing time 11 – 13 days
Post-treatment follow-up time 24 months
Overall duration of the study 72 months

3rd quarter 2017 Submission CPP and ANSM
Q1 2018 Initiation of the centers + Inclusion of the first patient
Q1 2022 Inclusion of the last patient
Q1 2024 Last follow-up visit
Q3 2024 Analysis and end-of-study report

VI.7. Toxicity assessment

The toxicity profile will be assessed using the NCI-CTCAE v.4.03 scale. Any toxicity, and its potential attributable to stereotactic radiation therapy, will be graded and recorded by the investigator. Particular attention will be paid to the evaluation of the following toxicities possibly related to stereotactics:

- Mucositis
- Difficulty in swallowing
- Dry mouth
- Mandibular osteonecrosis
- Soft tissue necrosis
- Epidermatitis
- Fibrosis.

VI.6.1 Acute toxicity

Acute toxicity will be assessed at each in-treatment visit and at each short-term follow-up visit (≤ 3 months after completion of radiotherapy). It will be assessed using the NCI-CTCAE v.4.03 scale.

VI.6.2 Late toxicity

Late toxicity will be assessed during each long-term follow-up visit (> 3 months from the end of radiotherapy), according to the NCI-CTCAE v.4.03 scale.

VI.8. Effectiveness evaluation

VI.7.1 Locoregional control

Evaluation of the tumor bed (clinical +/- nasofibroscopy) and cervical lymph nodes will be performed in accordance with the table of investigations and evaluations (see Table 1).

Evaluation by imaging (+/- panendoscopy) will be repeated as soon as appropriate in case of clinical doubt about tumor recurrence. Imaging evaluation will be systematic at 3 months, 1 year and 2 years after the end of radiotherapy.

- **Local recurrence**

Recurrence will be considered local if documented in the operative bed area. The diagnosis of local recurrence requires histological documentation. The date of recurrence will therefore be equal to the date of the biopsy that allowed histological confirmation.

- **Regional recidivism**

Recurrence will be considered regional if it occurs in the form of positive lymphadenopathy at the ipsilateral or contralateral cervical level

The diagnosis of a regional recurrence requires confirmation by imaging (CT scan and/or MRI and/or PET-FDG) and/or histological. The date of regional recurrence will be the date of the CT scan or MRI or PET – FDG highlighting the progression or the date of the biopsy in case of histological confirmation.

If histological confirmation of regional recurrence cannot be obtained, the diagnosis may be made based on the investigator's judgment.

VI.7.2 Remote Recidivism

The remote diagnosis of recurrence will be performed when one of the following criteria is met:

- Clear radiological evidence of distant metastasis on imaging (CT or MRI or PET – FDG)
- Positive biopsy (in case of radiological doubt or clinically suspected recurrence such as skin metastasis or palpable mass)

The date of distant recurrence will be the date of the CT scan or MRI or PET – FDG highlighting the metastases or the date of the biopsy in case of histological confirmation.

If histological confirmation of distant recurrence cannot be obtained, the diagnosis may be made based on the investigator's judgment.

VI.7.3 Second cancer

The diagnosis of a second cancer requires histopathological evidence of a new tumor mass outside the operative bed area, which is not consistent with the diagnosis of local recurrence or regional lymph node recurrence.

The date of diagnosis of the second cancer will be the date of histological confirmation, i.e. the date of the biopsy that led to the diagnosis.

VI.7.4 2-year recurrence-free survival (SSR)

SSR is defined as the time interval between the date of inclusion and the date of the first carcinologic event (local recurrence, lymph node recurrence, distant recurrence(s)) or death from any cause. Patients alive without disease recurrence (as defined above) will be censored on the date of the last follow-up visit.

VI.7.5 2-year overall survival (OS)

OS is defined as the time interval between the date of inclusion and the date of death from any cause. Live patients will be censored on the date of the last follow-up visit.

VI.9. Quality of life assessment

Quality of life (QoL) is assessed by the European *Organisation for Research and Treatment of Cancer (EORTC*) questionnaires QLQ-C30 and QLQ-H&N35, French version.

The QLQ-C30 questionnaire *(Appendix 3)* is a cancer-specific questionnaire that consists of 30 questions. Twenty-four questions participate in 9 multi-item scales: 5 functional scales (physical, role/position, cognitive, emotional, social), 3 symptom scales (fatigue, nausea/vomiting, pain) and one global condition scale. The remaining six questions are single-item scales assessing symptoms (dyspnea, insomnia, loss of appetite, constipation, diarrhea) and financial difficulties.

The QLQ-H&N35 questionnaire *(Appendix 3)* is a specific questionnaire for patients who have ENT cancer that consists of 35 questions. Twenty-four questions participate in 7 multi-item scales that assess ENT pain, swallowing, senses (taste and smell), speech, eating in society, social contacts and sexuality. The remaining 11 questions are uni-item scales describing specific concerns of patients with ENT cancer (dentition, mouth opening, dry mouth, sticky saliva, coughing, feeling unwell, taking painkillers, nutritional supplements, feeding tube, weight loss or gain).

The moments of collection of the quality of life questionnaires are:

- At the inclusion visit, i.e. after informed consent has been obtained and before the start of stereotactic radiotherapy
- At the 30±5-day follow-up visit after stereotactic radiotherapy
- At the follow-up visit 12 months±10 days after stereotactic radiotherapy
- At the 24-month follow-up visit±10 days post-radiotherapy

VII. BIOLOGICAL STUDY

For all patients included, a sample of their tumor taken during diagnosis or surgery will be retrieved. The analysis of the HPV (immunohistochemistry p16) status of the tumor will be performed in case it was not done by the center initially. These samples will be stored at the sponsor's premises with a view to possible future research.
The samples will be labelled with a unique code and will be kept under the responsibility of the sponsor until the publication of the results and no later than 15 years after the end of the research. The proponent will be responsible for the destruction of the samples at the end of the retention period.

VIII. TREATMENT UNDER STUDY: STEREOTACTIC RADIATION THERAPY

VIII.1. Description of the treatment

VIII.1.1 Infrastructure, equipment

The stereotactic radiotherapy technique will be left free depending on the equipment of the participating care institutions. Accelerators dedicated to stereotactic and equipped multi-purpose accelerators are permitted. Participating centres must comply with the rules and procedures of the Quality Assurance of Radiotherapy (see *Radiotherapy Guide* for details).

VIII.1.2 Quality assurance of radiotherapy

The evaluation of the quality of the treatment will be carried out through a radiotherapy quality assurance process including the validation of a test case (*Dummy run*) and a retrospective control of the files of all included patients (see *Radiotherapy Guide* for details).

- **Test case or *Dummy run***

This first step in the radiotherapy quality assurance process can be carried out as soon as it is feasible and before the first patient is included. This 2-step procedure will contain i) delineation and ii) dosimetry according to the protocol recommendations of a test case that will be provided. This test case will be reviewed centrally by the GORTEC Quality Assurance committee of experts. The participation of the centers will be confirmed or rediscussed, in particular depending on the compliance results of *Dummy run*.

- **Retrospective control**

Retrospective monitoring concerns all patients included in each center and will be carried out as quickly as possible up to a maximum of 30 days after the end of treatment to allow for possible major corrections if necessary for patients included later. For all patients, complete treatment plans, including diagnostic imaging, will be uploaded to a dedicated DICOM-RT platform for centralized review. Minor and major deviations for quality assurance are described in the *Radiotherapy Guide.*
It is essential to be able to provide:

- RT-doses, RT-structures and RT-images
- The compression method for repositioning (specific mask for stereotactic)
- The associated daily IGRT (*Image Guided Radiation Therapy*) method (depending on equipment and institutional habits)

VIII.1.3 Dental Care

All patients included will be required to have received a complete endooral and dental examination including a clinical and radiological examination. Tooth extractions will have to be performed if necessary. The interval between extractions and the start of radiotherapy will be **at least 10 days**. Appropriate dental care (including daily fluoroprophylaxis if necessary) will be recommended for all patients, at least from the follow-up period.

VIII.1.4 Patient Immobilization

All patients will be irradiated in the decubitus position. An immobilization procedure using compression systems adapted (personalized stereotactic masks) to the location and technique will be used to ensure that the accuracy and reproducibility of the patient's positioning during radiotherapy will be optimal.

VIII.1.5 Processing position and data acquisition

For the dosimetry scan, joint sections extending at least from the base of the skull to below the collarbones will be necessary. The thickness of the cuts will be a maximum of 2 mm. This dosimetric scanner will be performed in the treatment position with all the procedures and immobilization devices used on the treatment tables. It is recommended not to use injected imaging with contrast medium for dosimetry. On the other hand, this injection (in the absence of contraindications) is recommended in many cases for delineation. If to increase the contrast between the vessels and the peri-lesional soft tissues, a contrast agent is used according to institutional protocols, this imaging will be used as secondary imaging with registration with the imaging used for dosimetry.
A personalized transparent radio spacer that spreads the hard palate or tongue can be used according to the local habits of each center and according to the case.

VIII.1.6 Definition of volumes

VIII.1.6.1 Microscopic Target Volume (CTV) Delineation

Before starting the delineation, it will be necessary to analyze the preoperative data:

- ❖ Initial diagram of the tumor,
- ❖ Preoperative imaging (CT scan +/- MRI +/- PET-FDG),
- ❖ Endoscopy report,
- ❖ Operative report
- ❖ Anatomical and pathological report.

The patient will have been clinically re-evaluated (at most within 4 weeks prior to the start of radiotherapy) before the dosimetry CT scan because changes may appear between the surgery and the dosimetry scan. A realignment between the preoperative imaging and the dosimetry scanner can be performed to help delineate the CTV.

The CTV will correspond to the initial tumor bed including the positive or close margin with a margin of 5 to 10 mm depending on the anatomical barriers and the areas of infiltration. The indicative limits of the CTV by location are described in Table 2. In the case of flap reconstruction, the CTV will also include the normal/flap junction + 5 mm flap in front of the tumor bed. Peritumoral sutures with respect to mucosal healthy tissue will be included in the CTV. This requires a precise clinical examination before delineation to be able to report them on the dosimetry scanner.

VIII.1.6.2 Delineation of the Projected Target Volume (PTV)

The margins of the CTV to the PTV should take into account the uncertainties of the patient's position on the treatment table, the mobility of the tumour and the healthy organs that may change the position of the tumour, and the accuracy of the IGRT technique.

This margin will be defined by each participating centre based on their equipment, irradiation technique and experience. Typically, for patients immobilized by a stereotactic mask, with daily repositioning by IGRT, a margin of 2 mm appears to be suitable. A margin > to 3 mm is not recommended.

Table 2. Limits of the CTV according to location (to be adapted on a case-by-case basis)

Preoperative tumor localization	CTV Limits (for illustrative purposes)
All locations	The anterior and posterior boundaries should be adapted according to the limit of the initial lesion.
Movable tongue 1/3 middle and posterior	Medial: Up to midline<br>Lateral: in contact with the mandible<br>inferior: to the mylohyoid muscle
Movable tongue 1/3 anterior	Medial: Beyond the midline depending on the forward extension<br>Lateral: in contact with the mandible<br>Inferior: up to mylohyoid muscle
Floor 1/3 medium 1/3 posterior	Medial: 1 cm of movable tongue<br>Lateral: 5 mm mandible<br>Inferior: up to mylohyoid muscle
Front floor	Medial: 1 cm of mobile tongue exceeding the midline depending on the extension<br>Lateral: 5 mm mandible<br>Inferior: up to mylohyoid muscle
Inner side of the cheek	Lateral: 3 mm under the skin<br>Superior and inferior: gingival sulcus
Lower gum	Lateral: gingival-jugal groove over 1 cm<br>Medial: 5 mm floor<br>Inferior: mandible
Upper gum	Lateral: gingival-jugal groove over 1 cm<br>Medial: 1 cm hard palate<br>Upper: bone in its thickness
Hard palate	Medial: midline<br>Lateral: gingival-jugal groove over 1 cm<br>Upper: bone in its thickness
Anterior Abutment	Lateral: pharyngeal constrictor<br>Upper: mid-sail<br>Inferior and anterior: junction zone<br>Posterior: tonsil
Tonsil	Lateral: pharyngeal constrictor<br>Upper: mid-sail<br>Posterior: posterior pillar<br>Anterior: anterior pillar<br>Inferior: amygaloglossus and 3 folds
Veil	Inferior and lateral: 1/2 upper amygdala<br>Medial: beyond the uvula
Posterior Abutment	Lateral: pharyngeal constrictor<br>Upper: mid-sail<br>Bottom: Junction area<br>Anterior: Tonsil<br>Posterior and medial: Posterior pharyngeal wall over 1 cm
Posterior oropharyngeal wall	Lateral: posterior pillar<br>Medial: 0.5 to 1 cm beyond the midline<br>Posterior: pharyngeal constrictor
Language Basics	Lateral: tonsillagus<br>Median: Midline<br>Posterior: above the vallecula<br>Inferior: above the geniohyoid muscle

VIII.1.6.3 Delineation of organs at risk (OARs) and forecast OAR volumes (PRVs)

The delineation of the different OARs will be done according to Brouwer's recommendations *et al.*(44): medullary cord, medullary canal, brainstem, parotids, mandible, lips, pharyngeal constrictor muscles, submandibular glands, carotid arteries, cochlea, inner cheek surfaces, oral cavity and supraglottic larynx. Other normal structures or optimization volumes can be delineated to improve the planning optimization process. This will be left to the choice of the radiotherapist and the physicist.

For certain OARs (spinal cord cord, brainstem, cochleas and carotid arteries in particular) an additional margin may be added to the volume of the organ at risk to create a predictive volume of the organ at risk (PRV) taking into account the mobility of this organ and the imprecision in delineation and repositioning.

This margin will be selected by each participating centre based on their equipment, irradiation technique and experience. Typically, for patients immobilized by a stereotactic mask, with daily repositioning by IGRT, a margin of 1 mm appears to be appropriate.

A standardized nomenclature of target volumes, structures and organs at risk is proposed in Table 3, to which it is recommended to comply.

Table 3. The modalities of delineation of the different volumes.

Volume	Nomenclature	Delineation
CTV	CTV36Gy	Initial tumor bed including the positive or close margin + 5-10 mm. If flap, also include the junction of normal tissue to flap + 5 mm flap in front of the tumour bed. Peritumoral sutures in relation to mucosal healthy tissue will be included. See Table 2 for indicative limits of CTV based on the location of the initial tumor.
PTV	PTV36Gy	CTV + 2 mm (to be adapted according to the institution)
Spinal cord	Marrow	From the brainstem – spinal cord junction up to 10 cm below the PTV (recommended GRP of +1mm)
Spinal canal	Canalmed	From the brainstem – spinal cord junction up to 10 cm below the PTV (recommended GRP of +1mm)
Brain stem	Brainstem	Integral organ (+1mm recommended GRP)
Parotids	ParotideHL<br>ParotideCL	Organ in its entirety
Mandible	Mandible	Organ in its entirety
Lips	Lips	Organ in its entirety
Pharyngeal constrictor muscles	Constrictors	Organ in its entirety
Submandibular glands	SubmaxHL<br>SubmaxCL	Organ in its entirety
Carotid arteries	CarotidHL<br>CarotidCL	(recommended GRP of +1mm)
Cochleas	CochleaHL<br>CochleaCL	Integral organ (+1mm recommended GRP)
Inner cheek faces	JoueHL<br>JoueCL	Organ in its entirety
Mouth	Cavity	Organ in its entirety
Supraglottic Larynx	Larynx	Organ in its entirety

Legend: The delineation of the different SROs will be carried out according to the recommendations of Brouwer et al.(44).
Abbreviations: HL: Homolateral; CL: contralateral.

VIII.2. Doses, administration modalities and duration of treatment

VIII.2.1 Prescribing, Specification and Ratio of Dose to the VTP

A dose of 36Gy in 6 fractions delivered in 11-13 days seems to be the most appropriate regimen in this indication in terms of benefit/risk ratio (*see I.4. for details on the calculation of* BEDs). In the absence of a specific ICRU recommendation for stereotactic radiotherapy, the prescription is generally made to encompass at least 95% of the PTV in the prescribed physical dose, which in this case is 36Gy.
The dose will usually be prescribed on the usual isodose chosen with the stereotactic technique. Normalization on the maximum dose within the PTV varying with the stereotactic technique may be necessary.
Several types of prescriptions are authorized depending on the equipment and techniques of each center, in particular prescriptions such as RCMI or Cyberknife type (see below). The prescribed physical dose of PTV in the envelope is always 36Gy.

- ❖ **In RCMI type prescription**The prescription isodose of 36Gy will be 100% isodose to cover at least 95% of the PTV. The coverage of the PTV will be as homogeneous as possible due to the post-operative situation. It is recommended that 5% of the PTV (D5%) receive no more than 39.6Gy (110% of the prescribed physical dose of 36Gy) (45) and that 1cc of healthy tissue (outside the PTV) receives no more than 39.6Gy (110% of the prescribed physical dose of 36Gy).
- ❖ **As a prescription such as Cyberknife**, the prescription isodose of 36Gy will be determined to limit heterogeneities within the target volume as much as possible (classically isodose 80%) to cover at least 95% of the PTV. This implies that important heterogeneities are thus tolerated within the PTV but are not sought and should be as low as possible. The prescription must clearly measure the risk of a considerable increase in the dose delivered to a sensitive organ with a high probability of risk of complications (see Table 4 of the dose-volume objectives for planning). It is recommended that 20% of the PTV (D20%) receive no more than 39.6Gy (110% of the prescribed physical dose of 36Gy) (45) and that 1cc of healthy tissue (outside the PTV) receives no more than 39.6Gy (110% of the prescribed physical dose of 36Gy).

It should be noted that these prescription modalities are dependent on the stereotactic technique but also on the organs at risk and that the recording of deviations in terms of dose to the SROs is provided for in the protocol (see Table 4 of the dose-volume objectives for planning).
The conformance and homogeneity indexes will be reported in the eCRF.

Table 4. Dose-volume objectives for planning.

PTV or SRO/PRV	D95%	D min	D2%<br>D max	D5%<br>D20%	D avg<br>Average Dose	Dose/<br>volume
PTV prescription RCMI	36Gy	≥32.4Gy<br>(90% of 36Gy)	Dmax ≤ 45Gy<br>(≤25% of 36Gy)	D5% ≤ 39.6 Gy (110% of 36Gy)		
PTV prescription Cyberknife	36Gy	≥32.4Gy<br>(90% of 36Gy)	Dmax ≤ 45Gy<br>(≤25% of 36Gy)	D20% ≤ 39.6 Gy (110% of 36Gy)		
GRP Spinal cord			Dmax≤ 18 Gy			
PRV Spinal Canal			D2%≤ 22.5 Gy			
GRP Chiasma			D2%≤ 27 Gy			
PRV Optic nerves			D2%≤ 27 Gy			
GRP Brainstem			D2%≤ 27 Gy			
CL Paroid					≤ 5 Gy	
HL parotid					≤ 20 Gy	
Mandible			D2%≤ 36 Gy<br>(Mandible excluding PTV)			V29Gy<30%<br>V32Gy<16%
Supraglottic Larynx					≤ 28 Gy	
Submandibular gland CL					≤ 5 Gy	
Submandibular gland HL					≤ 26 Gy	
PRV Carotid Artery HL (excluding PTV)			D2%≤36 Gy			
GRP Cochlea HL			Dmax≤ 18 Gy			
GRP Cochlea CL			Dmax ≤ 5 Gy			
HL inner side of the cheek (excluding PTV°)					≤ 28 Gy	
CL inner cheek surface					≤ 5 Gy	
Oral cavity (excluding PTV)			D2%≤36 Gy		As low as possible	
Pharyngeal constrictor muscle					≤ 12 Gy	
Lips					≤ 5 Gy	

HL: ipsilateral; CL: contralateral.

Doses to SROs should be kept as low as possible.

VIII.2.2 Spreading

- Patients will preferably be treated with ***the first fraction delivered on Monday*.**
- The 6 fractions will be delivered in 11-13 days, 3 fractions per week.
- A minimum of 36 hours will be required between 2 fractions.

VIII.2.3 Treatment Planning

The stereotactic irradiation technique is left to the choice of the investigator according to the equipment available. The arrangement of the beams, the choice of energies, the planimetry according to the TPS, are left to the discretion of the investigators provided that they comply with the recommendations defined in the protocol in order to obtain an optimal dose distribution. The use of non-coplanar beams is permitted.

The dose-volume objectives for planning are described in Table 4.

VIII.2.4 Time Interval Between Surgery and Stereotactic Radiotherapy

The time interval between surgery of the primary tumor and the start of stereotactic radiotherapy will be ***less than 6 weeks, maximum 8 weeks***.

VIII.2.5 Checking positioning during stereotactic radiotherapy sessions

The positioning of the patients should be checked at each treatment session with an IGRT procedure adapted according to the institutional protocol according to the technique and the anatomical location of the volume treated. The corrections necessary for optimal repositioning will be systematically applied.

VIII.2.6 Interruptions or modifications of treatment

- No changes to target volume selection and delineation, dose prescribing, or spreading will be permitted (major deviations).
- The local investigators will take all appropriate measures to avoid any interruption and/or modification of the total dose delivered.

However, it is the responsibility of the local investigator to discontinue treatment in a duly justified manner if it is in the best interest of the patient. This interruption will be documented in the medical record and reported in the eCRF. ***In the event of a breakdown or overhaul of the accelerator or public holidays, all measures must be taken to avoid prolonging the spread of the treatment***.

IX. STATISTICAL CONSIDERATIONS

IX.1. Number of topics to include

To determine the number of patients needed, two objectives were considered: severe late toxicity at 2 years (primary outcome) and local control rate at 2 years (secondary outcome). The experimental design follows a one-step Fleming design with no intermediate analysis due to the primary endpoint which will be evaluated at 2 years (severe late toxicity).
The safety and efficacy profile of hypofractionated stereotactic radiotherapy of the operative bed is expected to be equivalent to other radiotherapy techniques (brachytherapy or normofractionated IMRT) (cf 3. Rationale of the study for details). In brachytherapy, the rate of late soft tissue ulceration is around 10-20% and the rate of bone necrosis is around 5-10%. We have decided: 1/ to accept a rate of less than 5% and 2/ to reject a rate of more than 15%. With a unilateral test for significance (α=0.05) with a power of 90% (β=0.10), the minimum number of patients to be included is 67. Hypofractionated stereotactic radiotherapy of the operative bed will then be considered an unacceptable treatment if 6/67 patients (9%) or more have severe late toxicity (grade $\geq$ 3).
The local control rate of hypofractionated stereotactic radiotherapy is expected to be equivalent to other radiotherapy techniques (brachytherapy or normofractionated IMRT). We have decided: (i) to accept a rate of more than 90% and (ii) to reject a rate of less than 80%. With a one-sided significance test (α=0.05) with a power of 80% (β=0.20), the minimum number of patients to be included is 83. A lower potency was decided for control as a secondary outcome. Hypofractionated stereotactic radiotherapy of the operative bed will then be considered an acceptable treatment if 72/83 patients (87%) or more are locally controlled at 2 years.

As a result of these results, and to compensate for possible loss to follow-up, a total of 90 patients will be included in this study.

As the primary objective of this study is estimated at 2 years, once 30 patients have been included (33% of inclusions), a review of all grade $\geq$ 3 toxicities will be communicated to the independent trial monitoring committee (IDSMC = *Independent Data and Safety Monitoring Committee*). The grade 4 toxicity rate is expected to be less than 10%. Inclusions will not be stopped during this evaluation.

IX.2. Data analysis: general

Patients will be described and compared between groups at baseline according to the following variables:

- Compliance with eligibility criteria
- Epidemiological characteristics
- Clinical and treatment characteristics.

A description of the deviations from the protocol, the patients distributed according to these deviations and the causes of abandonment will also be carried out. The number of patients included and the curve of inclusions will be presented by groups.

Continuous variables will be presented as a mean and standard deviation, subject to the normality of their distribution (Shapiro-Wilk test if necessary). In case of non-normality, they will be presented in the form of median, quartiles and extreme values. The qualitative variables will be expressed in terms of numbers and associated percentages. 95% confidence intervals (95% CI) will be reported. For each variable, the number of missing data will be specified. Graphical representations will be associated with these analyses as much as possible.

The estimate of the theoretical follow-up time will be the time difference between the date of inclusion and the date of the database freeze. The follow-up will also be estimated according to the reverse Kaplan Meier method for the observed follow-up. The parameters related to the time to events (SSR, OS...) as well as the time to deterioration of quality of life (scores) will be estimated according to the Kaplan-Meier method and described with a median confidence interval and rates at defined periods. Patients without specific events at the time of study termination (baseline freeze of study data) will be censored, for the "time to events" criteria, as of the date of the last update. Possible comparisons between independent groups will consider the log-rank test (univariate analysis) and the Cox model (multivariate analysis).

Tests will be bilateral, and a p< value of 0.05 will be considered statistically significant. As this trial is exploratory, the adjustment of the risk of error of the first kind will not be proposed systematically, but on a case-by-case basis in view of clinical and not only statistical considerations (48).

IX.3. Main analysis

The analysis of the main objective, i.e. the rate of severe toxicity of grade ≥ 3 to 2 years, with its 95% CI will be compared to 15% (with regard to the assumptions set in the context of the estimation of the number of subjects needed). If the 95% CI does not include this rate, the trial will be considered positive. For efficacy endpoints, 95% bilateral confidence intervals will be provided. Statistical analysis will be performed on an intention-to-treat basis.

An analysis of the predictive factors of severe toxicity of grade ≥ 3 to 2 years will be performed. A univariate analysis will be performed using the usual tests: Student's test or Mann-Whitney test if the conditions of the t-test are not met (normality, and homoscedasticity studied by the Fisher-Snedecor test) for quantitative variables and Chi2 test or Fisher's exact test for categorical variables. The multivariate analysis will consider a logistic regression model for binary dependent variable. Covariates will be retained based on the results of univariate analysis and their clinical relevance.

IX.4. Secondary analyses

QoL will be assessed by two validated questionnaires (EORTC QLQ-C30 and HN35) at different measurement times. Changes in quality of life will be studied in accordance with recent work (47). A difference of at least 10 points will be considered the smallest difference of clinical significance. Sensitivity analyses with a difference of at least 5 points will be performed.

For the different endpoints studied, a longitudinal study will be proposed by considering mixed models to take into account inter- and intra-patient variability ('random intercept' and 'slope' effects). These analyses will thus propose to study the (fixed) group effects (sex, age, anatomical pathological criteria...), evaluation time and their interaction while taking into account the center and subject effects as well as random in order to model inter- and intra-patient variability on the one hand and inter- and intra-center on the other hand. More precisely, to compare the means of the quantitative criteria between the inclusion and each measurement time, a linear mixed-effects model of the cLDA (constrained Longitudinal Data Analysis) type will be used. In this model, the variable to be explained includes both the baseline and post-inclusion values and the mean baseline difference between the groups is constrained to 0. This model makes it possible to calculate the changes between the inclusion and each follow-up time while adjusting on the values at inclusion. The difference between each group at each time is given through the interaction between the time and the randomization arm. The model will include different random effects: at the patient level (model with y-intercept and random slope allowing variation between patients in baseline values as well as changes in the outcome over time) and at the center level. The model parameters will be estimated using the Restricted Maximum Likelihood (REML) method.

IX.5. Method of accounting for missing, unused or invalid data

Particular attention will be paid to missing data. A sensitivity analysis will be performed to investigate the attrition bias, i.e. the quantity (level of attrition) and the nature (independence from the

randomization group) of the missing data in order to propose the most appropriate data imputation method (maximum bias, multiple imputation, Verbeke and Molenberghs estimate).

IX.6. Responsible for analysis

All statistical analyses will be performed with the *Stata software* (version 13, StataCorp, College Station, USA) in accordance with the recommendations of the *International Conference on Harmonization-Good Clinical Practice*. An analysis plan will be drawn up before the database is frozen.

The persons responsible for the statistical analysis are:
Ms Ioana MOLNAR (Tel: 04 73 27 80 75; ioana.molnar@clermont.unicancer.fr)
Mr. Bruno PEREIRA (Tel: 04 73 17 84 10; bpereira@chu-clermontferrand.fr)

X. INDEPENDENT OVERSIGHT COMMITTEE

The *Independent Data and Safety Monitoring Committee* (*IDSMC*) will conduct a review of all safety data from the trial and all events identified as requiring potential advice. It will be composed of 3 experts: 2 radiotherapists and 1 statistician/methodologist. The experts conducting this review will be selected for their expertise in clinical research and in the field of the study and will conduct an independent review. The IDSMC may propose modifications to the protocol (eligibility criteria, dose, etc.), premature termination of the trial, etc. Its opinion is advisory to the Promoter, who is responsible for making the final decision on the implementation of the recommendations proposed by this committee.
Since the primary objective of this study is evaluated at 2 years, once 30 patients have been included (33% of inclusions), a review of all grade ≥ 3 toxicities will be reported to the IDSMC. The grade 4 toxicity rate is expected to be less than 10%. Inclusions will not be stopped during this evaluation.

XI. SECURITY ASSESSMENT – ADVERSE EVENT MANAGEMENT

XI.1. Definitions

XI.1.1 Adverse Event

An adverse event (AE) is defined as any adverse event that occurs in a person who is engaged in research involving humans, whether or not the adverse event is related to the research or the product that is the subject of the research

XI.1.2 Serious adverse event or reaction

A serious event is an event:

- whose course is fatal,
- or that endangers the life of the person who is involved in the research,
- or that results in a significant or lasting disability or disability,
- or that causes hospitalization or a prolongation of hospitalization
- or that results in a congenital anomaly or malformation
- or any other event that does not meet the qualifications listed above, but may be considered "potentially serious", including certain biological abnormalities
- or medically relevant event in the judgment of the investigator

Certain circumstances requiring hospitalization do not fall under the criterion of seriousness: "hospitalization/prolongation of hospitalization" such as:

- Admission for social or administrative reasons
- Protocol-predefined hospitalization
- hospitalization for medical or surgical treatment scheduled prior to research
- Passage to day hospital

The expression "*which endangers the life of the person* " is reserved for an immediate life-threatening threat, at the time of the adverse event, regardless of the consequences of corrective or palliative therapy.

The term "*disability*" or "*incapacity*" refers to any clinically significant disability, whether temporary or permanent.

Deaths, regardless of their cause, including when they correspond to a progression of the disease being treated, are considered serious events.

Other events that do not meet the above qualifications may be considered "*potentially serious*", including certain biological abnormalities. The medical judgment of the investigator or sponsor may lead to the reporting of such events in the same manner as "serious" events.

Some events not to be considered serious in this study:

Disease progression should not be considered a SAE.

Due to the severity of the disease in this study, certain conditions defined as SAEs will be excluded from the SAE reporting procedure, namely:

- ❖ Scheduled hospitalization prior to the start of the trial
- ❖ Hospitalization or surgery related to the treatment of the disease
- ❖ Any event occurring between the signing of the consent and the performance of

stereotactic radiotherapy

- ❖ Deaths related to disease progression.

XI.1.3 Adverse Reaction

The adverse reaction of a search is any adverse event due to the research.
Serious adverse reactions are classified into the following subclasses:

- Expected adverse reactions

Expected serious adverse reaction : when already mentioned in the most recent version of the protocol. In this protocol, the expected side effects are those due to stereotactic radiotherapy:

Acute:

- ❖ Mucositis
- ❖ Epidermatitis
- ❖ Dysphagia that may require in some cases the placement of a feeding tube in the stomach
- ❖ Xerostomia (saliva may be thicker at first and then become rarer with dry mouth. Improvement in saliva can be seen up to two years after radiotherapy).
- ❖ Dysgeusia, ageusia
- ❖ Mucosal ulcerations

Late:

- ❖ Mandibular osteonecrosis (expected rate< 5-10%) (Special attention should be paid to dental care)
- ❖ Soft tissue necrosis (expected rate< 10-20%)
- ❖ Fibrosis

- Unexpected serious adverse reaction:

if its nature, severity or course differs from those described in the most recent version of the Investigator's Brochure or the Summary of Product Characteristics for a medicinal product or the Instruction Leaflet for a medical device.

XI.1.3 Developments

<u>New fact</u>: any new data that may lead to a re-evaluation of the benefit-risk balance of the research or the product being researched, to changes in the use of that product, in the conduct of the research, or

in documents relating to the research, or to the suspension or interruption or modification of the protocol of the research or similar research.

For example, we can mention:

- serious adverse reactions that are more frequent;
- the results of interim analyses, when they are relevant to the safety of people (in particular insufficient effectiveness);
- serious adverse events related to clinical trial procedures;
- insufficient efficacy in life-threatening diseases;
- the results of animal studies that would provide new information on the safety of the product;
- and in general, any new information that may lead to a re-evaluation, in an unfavourable direction, of the risk/benefit ratio of the research.

Any new fact concerning the research (or the product used) and likely to affect the safety of the persons who take part in the research will be the subject of appropriate urgent safety measures and information without delay by the Promoter to the competent authority and the Committee for the Protection of Persons.

XI.1.4 Other event to be considered serious: pregnancy/breastfeeding

In general, pregnancy and breastfeeding are exclusion criteria from the study. If pregnancy occurs during the study, the patient should be discharged from the study immediately. The patient should be monitored throughout her pregnancy and after the birth of the child.

XI.2. Reporting of serious adverse events

XI.2.1 Responsibility of the Investigator

For each type of research involving humans, the investigator records the adverse events or adverse analytical findings defined in the protocol as decisive for the safety assessment, keeps a paper trail and notifies them to the sponsor.
The investigator is responsible for reporting all adverse events in the case report form.
The severity or grade of the event should be assessed according to the toxicity criteria NCI-CTCAE-v.4.03 *(Appendix 1).*

The investigator shall inform the sponsor of all serious adverse events that have occurred in participants, unless otherwise provided for in the protocol.

The investigator is responsible for the appropriate medical follow-up of patients until the resolution or stabilization of the event or until the patient's death. This may sometimes mean that this follow-up continues after the patient has been discharged from the trial.

It transmits the additional information to the study's pharmacovigilance using a SAE reporting form.

It also transmits the latest follow-up to the resolution or stabilisation of the SAE.

It keeps the documents concerning the presumed adverse event in order to allow, if necessary, to complete the information previously transmitted.

It responds to requests for additional information from pharmacovigilance in order to document the initial observation and implements the SPONSOR's decisions in relation to SAEs.

The investigator shall notify the sponsor without delay from the day on which it becomes aware of any serious adverse event that has occurred in any patient included

- - for the entire duration of the study (up to 2 years after the end of stereotactic radiotherapy),
- After the trial has been stopped, regardless of the time limit, provided that no cause other than research can reasonably be incriminated,

on a "Serious Adverse Event" form indicating the date of occurrence, intensity, relationship with the treatment (or research) evaluated, and follow-up.

The narrative report must be completed and sent to the promoter as soon as new relevant information is obtained. Depending on the nature and severity of the event, copies of the patient's anonymized medical record may be attached, as well as the results of laboratory analyses.

When a serious adverse event persists at the end of the study, the investigator will follow the patient until the event is considered resolved.

XI.2.2 Methods for the collection and evaluation of adverse events

All serious and non-serious ARs should be reported in the case report form on the "Adverse events" page, indicating that they are an SAE (if applicable).

The following criteria must be recorded for each event in the eCRF:

- ❖ A description of the AR in standard medical terminology, not as reported by the patient
- ❖ The start date
- ❖ The recovery date
- ❖ The grade assessed by the investigator according to the definitions of the NCI-CTCAE, Version 4.03 *(Appendix 1)*
- ❖ The causal relationship between the event and the research, as assessed by the investigator:

- Linked to a pre-existing or underlying pathology
- Research-related (stereotactic radiotherapy)
- Related to other treatments (specify products)
- Other (e.g. accident, new pathology)

- Measure taken concerning stereotactic radiotherapy (none, discontinuation, dose reduction, temporary interruption, etc.).
- Another action.
- Evolution according to the following definitions:
  - Recovery with sequelae
  - Recovery without sequelae
  - Improvement
  - Persistence
  - Aggravation
  - Death
  - Unknown
- Serious: yes or no

XI.2.3 Dealing with serious adverse events

In the event of the occurrence of any clinical adverse event or abnormal laboratory value, considered serious whether or not attributable to the research and which occurs during the study (from the signing of the informed consent until 2 years after the end of radiotherapy). The investigator must inform the Sponsor **without delay** by sending a detailed written report using the Serious Adverse Event reporting form (for an "initial" SAE or for a "follow-up" of information on a previous SAE) by email or fax to the pharmacist in charge of pharmacovigilance at the Jean Perrin Center. Immediate or follow-up reports must identify the patient by the same code number.

Contact the promoter:

SOPHIE LEVESQUE
sophie.levesque@clermont.unicancer.fr
Fax: 04 73 27 80 29
Tel: 04 63 66 33 47

The case will be processed at the Jean Perrin Centre by the Pharmacovigilance Unit.

The Sponsor should ensure that all regulatory obligations regarding the reporting of an SAE have been complied with in accordance with local regulations and that the investigator has provided all necessary additional information. The "expected" nature of the event is verified in the relevant Product Information.

The investigator should also attach to the serious adverse event report, whenever possible:

- A copy of the report of hospitalization or extension of hospitalization,
- A copy of the autopsy report (if applicable),
- A copy of all the results of any further examinations performed, including the relevant negative results, together with the normal values of the laboratory,
- Any other document that it deems useful and relevant.

All these documents must be anonymized (a code will be assigned to each patient included in the study). Additional information may be requested (by fax, telephone or during a visit) by the monitor and/or pharmacovigilance

XI.2.4 Sponsor's Liability

In accordance with the Public Health Code relating to research on human persons, all suspicions of unexpected serious adverse reactions that could lead to death or life-threatening prognosis will be the subject of a declaration by the Sponsor to the ANSM:

- without delay (from the day on which the Promoter becomes aware of it)
- within 15 days of the occurrence of the event for the other severity criteria

The Sponsor will also send all study investigators a copy of any serious unexpected adverse reactions related to the trial.

The sponsor will decide on the meaning of the serious adverse events it reports and the consequences it draws from them, particularly with regard to the conduct of the research.

The sponsor will also decide on the imputability of the adverse event.

Predictability will be determined by the proponent.

The sponsor shall maintain detailed records of all adverse events reported to it by the investigator(s).

Once a year or on request, the promoter will send the ANSM and the CPP an annual safety report taking into account all available safety information.

The sponsor will also provide the study investigators with any information that may affect the safety of individuals.

XII. REPORT

The database will be created by GORTEC from the CLEANWEB software. The database will be hosted by the GORTEC server in Tours. The data will be backed up in real time and then archived daily.

The data will be entered via the internet after logging into the database with a username and password specific to each user, and giving them certain viewing and modification rights according to their profile. The data entry will be done in SSL (secure) mode, directly by an Internet browser (INTERNET EXPLORER, FIREFOX, CHROME, etc.), the data transmitted being encrypted. The history of each piece of data (with all changes, user name and date modified) can be viewed (AuditTrail). CLEANWEB complies with the Food and Drug Administration's 21 CFR Part 11 standard, as well as the standard for the security of computerized systems.

Data warnings can be displayed directly during entry to reduce errors. Consistency tests will be run either directly under CLEANWEB or under SAS after data extraction. They will give rise to the issuance of queries.

Once entered, the data can be exported in SAS, SPSS, STATVIEW, EXCEL, TXT format.

All the information required by the protocol must be recorded in the electronic CRF provided for this purpose and an explanation must be provided for each missing data.

XIII. RIGHT OF ACCESS TO SOURCE DATA AND DOCUMENTS

XIII.1. Access to data

The sponsor is responsible for obtaining the agreement of all parties involved in the research to ensure direct access to all research locations, source data, source documents and reports for the purpose of quality control and audit by the sponsor.

The investigators will make available the documents and individual data strictly necessary for the monitoring, quality control and audit of research on the human person, available to persons with access to these documents in accordance with the legislative and regulatory provisions in force (Articles L.1121-3 and R.5121-13 of the Public Health Code).

XIII.2. Source data

Source documents, being defined as any original document or object that proves the existence or accuracy of a data or fact recorded during the clinical study, will be kept for 15 years by the investigator or by the hospital in the case of a hospital medical record.

XIII.3. Data Privacy

In accordance with the provisions concerning the confidentiality of data to which persons responsible for quality control of research involving human persons have access (Article L.1121-3 of the Public

Health Code), in accordance with the provisions relating to the confidentiality of information concerning, in particular, the nature of the investigational medicinal products, the trials, the persons who lend themselves to them and the results obtained (Article R. 5121-13 of the Public Health Code), Persons with direct access shall take all necessary precautions to ensure the confidentiality of information relating to investigational medicinal products, trials, persons who are eligible for them, and in particular as regards their identity and the results obtained.

These persons, in the same way as the investigators themselves, are subject to professional secrecy (under the conditions defined by Articles 226-13 and 226-14 of the Criminal Code).

During or after human research, the data collected on suitable individuals and transmitted to the sponsor by the investigators (or any other specialized stakeholders) will be anonymized.

They must not under any circumstances display the names of the persons concerned or their addresses in plain text.

On the CRF and other documents used in the study, patients should not be identified by name but by a code. The investigator will keep a document where the patients' identities correspond to their code number.

The sponsor will ensure that each person who participates in the research has given his or her written consent for access to the individual data concerning him or her that is strictly necessary for the quality control of the research.

XIV. QUALITY CONTROL AND ASSURANCE

XIV.1. Investigator and sponsor engagement

The investigator undertakes to ensure that this study is carried out in accordance with the Public Health Act No. 2004-806 of 9 August 2004 concerning research on human persons, the implementing decree No. 2006-477 of 26/04/2006 amending Chapter I of Title II of Book 1^{of} Part One of the Public Health Code relating to research on the human person as well as the decrees in force.

The rules of good clinical practice (GCP) for research on human persons on medicinal products for human use, referred to in Article L. 1121-3 of the Public Health Code and the Decree of 24 November 2006, will also be applied.

The investigator is also committed to working in accordance with the Declaration of Helsinki of the World Medical Association (Fortaleza 2013, revised).

The study will be carried out in accordance with:

- ❖ The Jardé Law No. 2012-300 of 12 March 2012 (implementing decree of 16 November 2016 on research involving human beings)

- Regulation (EU) No 536/2014 of the European Parliament and of the Council of 16 April 2014 on clinical trials
- The Huriet Law (No. 88-1138) of 20 December 1988 on the protection of persons and amended by the Law of 9 August 2004 (No. 2004-806)
- The Data Protection Act No. 78-17 of 6 January 1978 amended by Law No. 2004-801 of 6 August 2004 on the protection of individuals with regard to the processing of personal data)
- The Bioethics Law No. 2011-814 of July 7, 2011
- Good Clinical Practice of 24 November 2006

XIV.2. Quality Assurance

A Clinical Research Associate (CRA) mandated by the sponsor will ensure the proper performance of the study, the collection of the data generated in writing, their documentation, recording and reporting, in accordance with the Standard Operating Procedures applied within the Jean Perrin Center and in accordance with Good Clinical Practices as well as the legislative and regulatory provisions in force. The monitoring of the data will be carried out by GORTEC.

XIV.3. Quality Control

The investigator guarantees the authenticity of the data collected in the study and accepts the legal provisions authorizing the study sponsor to implement quality control.

The coordinating investigator and the associate investigators therefore agree to make themselves available during the Quality Control visits carried out at regular intervals by the Clinical Research Associate. During these visits, the following elements will be reviewed:

- Informed consent
- Compliance with the study protocol and the procedures defined therein
- Quality of the data collected in the case report form: accuracy, missing data, consistency of the data with the "source" documents (medical records, appointment books, originals of laboratory results, etc.)

XV. ETHICAL CONSIDERATIONS

XV.1. Committee for the Protection of Persons

The protocol as well as the information and consent form will be submitted to the Committee for the Protection of Persons for its opinion and for authorisation to the ANSM.

XV.2. Patient Information and Written Informed Consent Form

The investigator is responsible for ensuring that each patient gives written consent to participate in the trial.

The investigator will explain to the patient the nature of the trial, its purpose, its methodology, the expected duration as well as the expected benefits, constraints and possible disadvantages. A "patient information leaflet" will be provided to each patient on the same sheet as the consent.

Patients will be informed of anonymity and their privacy rights, but will be aware that the trial data will be submitted to the sponsor as well as the Supervisory Authorities for review and evaluation.

Patients will be given the opportunity to ask questions and will be informed of their right to withdraw from the trial, at any time, without incurring any liability. After these explanations, the patient will voluntarily sign the "Patient Consent" document.

After reading the information leaflet, the patient, if he or she agrees to participate, must date and sign the consent form in two original copies. This consent must also be signed by the investigator. One original must be archived by the investigator and the other given to the research participant.

The information leaflet and the patient consent form have been proofread by the patient committee of the National League Against Cancer.

XV.3. Amendments to the Protocol

The changes made to the protocol will have to be qualified as substantial or not.

Depending on their nature, they will be the subject of a new opinion from the Committee for the Protection of Persons and/or an authorisation from the competent authority.

All investigators will acknowledge receipt of changes to the protocol and confirm by signing on the approval page of the new version of the protocol that they intend to abide by the terms of the protocol.

XVI. DATA PROCESSING AND RETENTION OF DOCUMENTS AND DATA RELATING TO RESEARCH

XVI.1. Data collection and processing

Data management will be carried out by GORTEC on the CLEANWEB software.

All statistical analyses will be performed with the *Stata software* (version 13, StataCorp, College Station, USA) in accordance with the recommendations of the *International Conference on Harmonization-Good Clinical Practice*. An analysis plan will be drawn up before the database is frozen.

XVI.2. CNIL

This study is part of the "Reference Methodology" (MR-001) in application of the provisions of the law of 6 August 2004 on the protection of individuals with regard to the processing of personal data and amending the law of 6 January 1978 on information technology, files and freedoms. This change was approved by decision of 5 January 2006, which was amended by deliberation no. 2016-262 of 21 July 2016. The Jean Perrin Center, sponsor of the study, signed a commitment to comply with this "Reference Methodology" on December 13, 2006. GORTEC, responsible for data management, also signed a commitment to comply with this "Reference Methodology" on June 4, 2007.

XVI.3. Archiving

The following documents will be archived by the name of the study until the end of the period of practical usefulness (period of recruitment and follow-up of patients + the time required for data analysis).

These documents are:

- Protocol and annexes, possible amendments
- Signed original information forms and consents
- Individual data (authenticated copies of raw data)
- Follow-up documents
- Statistical analyses
- Final report of the study

At the end of the period of practical usefulness, all the documents to be archived will be placed under the responsibility of the Promoter for 15 years after the end of the study in accordance with institutional practices.

No displacement or destruction may be carried out without the agreement of the Promoter. At the end of the 15 years, the developer will be consulted for destruction. All data, documents and reports may be subject to audit or inspection.

XVII.INSURANCE

In accordance with the regulatory provisions, the Jean Perrin Centre, in its capacity as promoter, has taken out civil liability insurance to cover any damage resulting from the research with AXA. The contract number is 36376041272087.

It should be noted that non-compliance with the legal conditions of the research (absence of an opinion from the CPP, lack of authorization from the ANSM, non-consent of the person, continuation of a suspended or prohibited research) is an exclusion clause from the guarantee.

XVIII. COMMUNICATION - PUBLICATION RULES

Data will only be disclosed after prior joint agreement of the investigator and the sponsor. The results will be the subject of communications and publications.

At the end of the trial, a report will be written by the sponsor and the principal investigator. No publication or presentation of the results of this trial may be made without the agreement of the investigators and coordinators. If this trial results in a publication, each investigator may be an author in an order determined by the number of evaluable included patients (usable records**).** The manuscript is signed by the principal investigator (first author) who will manage the correspondence. The statistician of the study will be a co-author of the publication. The bibliography will follow the Vancouver Rules. The co-coordinators may, if necessary, present the results at congresses in the same way as the principal investigator. The position of last or co-last author will be awarded to the co-principal investigator. The coordinator for the intergroup will be co-first author or last/co-last author. A representative of GETTEC and GERCOR will be associated with the publication.

All publications will be the subject of an information/presentation at the meetings of GORTEC, GETTEC and GERCOR. Any publication should mention that it is a study by the Promoter "GORTEC", carried out in partnership with GETTEC and GERCOR.

XIX. BIBLIOGRAPHY

1. Warnakulasuriya S. Global epidemiology of oral and oropharyngeal cancer. Oral Oncol. May 2009; 45(4-5):309-16.

2. Eckel HE, Volling P, Pototschnig C, Zorowka P, Thumfart W. Transoral laser resection with staged discontinuous neck dissection for oral cavity and oropharynx squamous cell carcinoma. Laryngoscope. Jan 1995; 105(1):53-60.

3. Ferlito A, Silver CE, Rinaldo A. Elective management of the neck in oral cavity squamous carcinoma: current concepts supported by prospective studies. Br J Oral Maxillofac Surg. Jan 2009; 47(1):5-9.

4. Duvvuri U, Simental AA, D'Angelo G, Johnson JT, Ferris RL, Gooding W, et al. Elective neck dissection and survival in patients with squamous cell carcinoma of the oral cavity and oropharynx. Laryngoscope. Dec 2004; 114(12):2228-34.

5. Capote A, Escorial V, Muñoz-Guerra MF, Rodríguez-Campo FJ, Gamallo C, Naval L. Elective neck dissection in early-stage oral squamous cell carcinoma--does it influence recurrence and survival? Head Neck. Jan 2007; 29(1):3-11.

6. Meier JD, Oliver DA, Varvares MA. Surgical margin determination in head and neck oncology: current clinical practice. The results of an International American Head and Neck Society Member Survey. Head Neck. Nov 2005; 27(11):952-8.

7. Bradley PJ, MacLennan K, Brakenhoff RH, Leemans CR. Status of primary tumour surgical margins in squamous head and neck cancer: prognostic implications. Curr Opin Otolaryngol Head Neck Surg. Apr 2007; 15(2):74-81.

8. Gomez DR, Zhung JE, Gomez J, Chan K, Wu AJ, Wolden SL, et al. Intensity-modulated radiotherapy in postoperative treatment of oral cavity cancers. Int J Radiat Oncol Biol Phys. 15 March 2009; 73(4):1096-103.

9. Hinerman RW, Mendenhall WM, Morris CG, Amdur RJ, Werning JW, Villaret DB. Postoperative irradiation for squamous cell carcinoma of the oral cavity: 35-year experience. Head Neck. Nov 2004; 26(11):984-94.

10. Zelefsky MJ, Harrison LB, Fass DE, Armstrong JG, Shah JP, Strong EW. Postoperative radiation therapy for squamous cell carcinomas of the oral cavity and oropharynx: impact of therapy on patients with positive surgical margins. Int J Radiat Oncol Biol Phys. Jan 1993; 25(1):17-21.

11. Beitler JJ, Smith RV, Silver CE, Quish A, Deore SM, Mullokandov E, et al. Close or positive margins after surgical resection for the head and neck cancer patient: the addition of brachytherapy improves local control. Int J Radiat Oncol Biol Phys. 15 Jan 1998; 40(2):313-7.

12. Jäckel MC, Ambrosch P, Christiansen H, Martin A, Steiner W. Value of postoperative radiotherapy in patients with pathologic N1 neck disease. Head Neck. July 2008; 30(7):875-82.

13. Lapeyre M, Coche-Dequéant B, Moreira J-F, Le Bourhis J, Peiffert D. [Brachytherapy for head and neck cancers]. Cancer Radiother. Apr 2013; 17(2):130-5.

14. Mazeron J-J, Ardiet J-M, Haie-Méder C, Kovács G, Levendag P, Peiffert D, et al. GEC-ESTRO recommendations for brachytherapy for head and neck squamous cell carcinomas. Radiother Oncol. May 2009; 91(2):150-6.

15. Strnad V. Treatment of oral cavity and oropharyngeal cancer. Indications, technical aspects, and results of interstitial brachytherapy. Strahlenther Onkol. Nov 2004; 180(11):710-7.

16. Goineau A, Piot B, Malard O, Ferron C, Lisbona A, Cassagnau E, et al. Postoperative interstitial brachytherapy for resectable squamous cell carcinoma of the tongue. Brachytherapy. Feb 2015; 14(1):71-6.

17. Lapeyre M, Bollet MA, Racadot S, Geoffrois L, Kaminsky M-C, Hoffstetter S, et al. Postoperative brachytherapy alone and combined postoperative radiotherapy and brachytherapy boost for squamous cell carcinoma of the oral cavity, with positive or close margins. Head Neck. March 2004; 26(3):216-23.

18. Lapeyre M, Hoffstetter S, Peiffert D, Guérif S, Maire F, Dolivet G, et al. Postoperative brachytherapy alone for T1-2 N0 squamous cell carcinomas of the oral tongue and floor of mouth with close or positive margins. Int J Radiat Oncol Biol Phys. 1 August 2000; 48(1):37-42.

19. Strnad V, Lotter M, Kreppner S, Fietkau R. Interstitial pulsed-dose-rate brachytherapy for head and neck cancer--Single-institution long-term results of 385 patients. Brachytherapy. Dec 2013; 12(6):521-7.

20. Chen P-Y, Chen HHW, Hsiao J-R, Yang M-W, Hsueh W-T, Tasi S-T, et al. Intensity-modulated radiotherapy improves outcomes in postoperative patients with squamous cell carcinoma of the oral cavity. Oral Oncol. August 2012; 48(8):747-52.

21. Chen AM, Farwell DG, Luu Q, Chen LM, Vijayakumar S, Purdy JA. Marginal misses after postoperative intensity-modulated radiotherapy for head and neck cancer. Int J Radiat Oncol Biol Phys. 1 August 2011; 80(5):1423-9.

22. Geretschläger A, Bojaxhiu B, Crowe S, Arnold A, Manser P, Hallermann W, et al. Outcome and patterns of failure after postoperative intensity modulated radiotherapy for locally advanced or high-risk oral cavity squamous cell carcinoma. Radiat Oncol. 2012;7:175.

23. Collan J, Lundberg M, Vaalavirta L, Bäck L, Kajanti M, Mäkitie A, et al. Patterns of relapse following surgery and postoperative intensity modulated radiotherapy for oral and oropharyngeal cancer. Acta Oncol. Oct 2011; 50(7):1119-25.

24. Chan AK, Huang SH, Le LW, Yu E, Dawson LA, Kim JJ, et al. Postoperative intensity-modulated radiotherapy following surgery for oral cavity squamous cell carcinoma: patterns of failure. Oral Oncol. March 2013; 49(3):255-60.

25. Gevaert T, Verellen D, Engels B, Depuydt T, Heuninckx K, Tournel K, et al. Clinical evaluation of a robotic 6-degree of freedom treatment couch for frameless radiosurgery. Int J Radiat Oncol Biol Phys. 1 May 2012; 83(1):467-74.

26. Guinement L, Marchesi V, Veres A, Lacornerie T, Buchheit I, Peiffert D. [Development of external quality control protocol for CyberKnife beams dosimetry: preliminary tests multicentre]. Cancer Radiother. August 2013; 17(4):288-96.

27. Benhaïm C, Lapeyre M, Thariat J. [Stereotactic irradiation in head and neck cancers]. Cancer Radiother. August 2014; 18(4):280-96.

28. Lo SS, Fakiris AJ, Chang EL, Mayr NA, Wang JZ, Papiez L, et al. Stereotactic body radiation therapy: a novel treatment modality. Nat Rev Clin Oncol. Jan 2010; 7(1):44-54.

29. Lartigau EF, Tresch E, Thariat J, Graff P, Coche-Dequeant B, Benezery K, et al. Multi institutional phase II study of concomitant stereotactic reirradiation and cetuximab for recurrent head and neck cancer. Radiother Oncol. Nov 2013; 109(2):281-5.

30. Vargo JA, Ferris RL, Clump DA, Heron DE. Stereotactic Body Radiotherapy as Primary Treatment for Elderly Patients with Medically Inoperable Head and Neck Cancer. Front Oncol [Internet]. 2014 Aug 11 [cited 2016 Jan 6];4. Available on: http://www.ncbi.nlm.nih.gov/pmc/articles/PMC4128217/

31. Heron DE, Ferris RL, Karamouzis M, Andrade RS, Deeb EL, Burton S, et al. Stereotactic body radiotherapy for recurrent squamous cell carcinoma of the head and neck: results of a phase I dose-escalation trial. Int J Radiat Oncol Biol Phys. 1 Dec 2009; 75(5):1493-500.

32. Wang K, Heron DE, Clump DA, Flickinger JC, Kubicek GJ, Rwigema J-CM, et al. Target delineation in stereotactic body radiation therapy for recurrent head and neck cancer: a retrospective analysis of the impact of margins and automated PET-CT segmentation. Radiother Oncol. Jan 2013; 106(1):90-5.

33. Rwigema J-CM, Heron DE, Ferris RL, Andrade RS, Gibson MK, Yang Y, et al. The impact of tumor volume and radiotherapy dose on outcome in previously irradiated recurrent squamous cell carcinoma of the head and neck treated with stereotactic body radiation therapy. Am J Clin Oncol. August 2011; 34(4):372-9.

34. Vargo JA, Kubicek GJ, Ferris RL, Duvvuri U, Johnson JT, Ohr J, et al. Adjuvant stereotactic body radiotherapy±cetuximab following salvage surgery in previously irradiated head and neck cancer. Laryngoscope. July 2014; 124(7):1579-84.

35. Vargo JA, Heron DE, Ferris RL, Rwigema J-CM, Kalash R, Wegner RE, et al. Examining tumor control and toxicity after stereotactic body radiotherapy in locally recurrent previously irradiated head and neck cancers: implications of treatment duration and tumor volume. Head Neck. Sep 2014; 36(9):1349-55.

36. Vargo JA, Ferris RL, Ohr J, Clump DA, Davis KS, Duvvuri U, et al. A prospective phase 2 trial of reirradiation with stereotactic body radiation therapy plus cetuximab in patients with previously irradiated recurrent squamous cell carcinoma of the head and neck. Int J Radiat Oncol Biol Phys. 1 March 2015; 91(3):480-8.

37. Al-Mamgani A, Tans L, Teguh DN, van Rooij P, Zwijnenburg EM, Levendag PC. Stereotactic body radiotherapy: a promising treatment option for the boost of oropharyngeal cancers not suitable for brachytherapy: a single-institutional experience. Int J Radiat Oncol Biol Phys. 15 March 2012; 82(4):1494-500.

38. Al-Mamgani A, Van Rooij P, Sewnaik A, Mehilal R, Tans L, Verduijn GM, et al. Brachytherapy or stereotactic body radiotherapy boost for early-stage oropharyngeal cancer: comparable outcomes of two different approaches. Oral Oncol. Oct 2013; 49(10):1018-24.

39. Thariat J, Poissonnet G, Marcy P-Y, Lattes L, Butori C, Guevara N, et al. Effect of surgical modality and hypofractionated split-course radiotherapy on local control and survival from sinonasal mucosal melanoma. Clin Oncol (R Coll Radiol). Nov 2011; 23(9):579-86.

40. Troussier I, Baglin A-C, Marcy P-Y, Even C, Moya-Plana A, Krengli M, et al. [Mucosal melanomas of the head and neck: State of the art and current controversies]. Bull Cancer. June 2015; 102(6):559-67.

41. Wu AJ, Gomez J, Zhung JE, Chan K, Gomez DR, Wolden SL, et al. Radiotherapy after surgical resection for head and neck mucosal melanoma. Am J Clin Oncol. June 2010; 33(3):281-5.

42. Fowler JF. Is there an optimum overall time for head and neck radiotherapy? A review, with new modelling. Clin Oncol (R Coll Radiol). Feb 2007; 19(1):8-22.

43. Fowler JF. Optimum overall times II: Extended modelling for head and neck radiotherapy. Clin Oncol (R Coll Radiol). March 2008; 20(2):113-26.

44. Brouwer CL, Steenbakkers RJHM, Bourhis J, Budach W, Grau C, Grégoire V, et al. CT-based delineation of organs at risk in the head and neck region: DAHANCA, EORTC, GORTEC, HKNPCSG, NCIC CTG, NCRI, NRG Oncology and TROG consensus guidelines. Radiother Oncol. Oct 2015; 117(1):83-90.

45. Quan K, Xu KM, Zhang Y, Clump DA, Flickinger JC, Lalonde R, et al. Toxicities Following Stereotactic Ablative Radiotherapy Treatment of Locally-Recurrent and Previously Irradiated Head and Neck Squamous Cell Carcinoma. Semin Radiat Oncol. Apr 2016; 26(2):112-9.

46. French Society of Otorhinolaryngology and Face and Neck Surgery (SFORL). Update of the 2005 recommendation on post-treatment follow-up of squamous cell carcinomas of adult VADS. 2016.

47. Bonnetain F, Fiteni F, Efficace F, Anota A. Statistical Challenges in the Analysis of Health-Related Quality of Life in Cancer Clinical Trials. J Clin Oncol. June 1, 2016; 34(16):1953-6.

48. Feise RJ. Do multiple outcome measures require p-value adjustment? BMC Med Res Methodol. 2002 Jun 17;2:8.

XX. LIST OF APPENDICES

APPENDIX 1: Rating of adverse events according to NCI-CTCAE V.4.03 criteria

National Cancer Institute - Common Terminology Criteria for Adverse Events

Refer to the CTCAE Toxicity Evaluation Scale version 4.03 which can be downloaded from the NCI website

Cancer Therapy Evaluation Program

http://evs.nci.nih.gov/ftp1/CTCAE/CTCAE_4.03_2010-06-14_QuickReference_5x7.pdf

Common Terminology Criteria for Adverse Events v.4.03 (NCI-CTCAE)

ANNEX 2: Assessment of general condition according to the Karnofsky classification and the WHO scale

	Karnofsky scale	WHO scale
Normal, no complaints	**100**	**0**
Normal activity.<br>Minor sign or symptoms of the disease.<br><br>Normal activity with effort.	**90**<br><br><br>**80**	**1**
Able to take care of themselves, but unable to have a normal activity or work.<br><br>Occasionally requires help, but able to provide for most of its needs	**70**<br><br><br>**60**	**2**
Requires frequent medical help and care.<br><br>Requires medical attention and significant assistance.	**50**<br><br>**40**	**3**
Severely limited, bedridden. Indication for hospitalization, although death is not imminent.<br><br>Seriously injured. Hospitalization necessary. Symptomatic treatment required.<br>Requires medical attention and significant assistance.	**30**<br><br><br>**20**	**4**

APPENDIX 3: EORTC QLQ-C30 and H&N35 Questionnaires

EORTC QLQ-C30 (version 3)

Nous nous intéressons à vous et à votre santé. Répondez vous-même à toutes les questions en entourant le chiffre qui correspond le mieux à votre situation. Il n'y a pas de "bonne" ou de "mauvaise" réponse. Ces informations sont strictement confidentielles.

Merci de préciser:

Vos initiales: |_|_|_|_|

Date de naissance (jour/mois/année): |_|_|_|_|_|_|_|_|

La date d`aujourd`hui (jour/mois/année): 31 |_|_|_|_|_|_|_|_|

	Pas du tout	Un peu	Assez	Beaucoup
1. Avez-vous des difficultés à faire certains efforts physiques pénibles comme porter un sac à provision chargé ou une valise?	1	2	3	4
2. Avez-vous des difficultés à faire une longue promenade?	1	2	3	4
3. Avez-vous des difficultés à faire un petit tour dehors?	1	2	3	4
4. Etes-vous obligée de rester au lit ou dans un fauteuil pendant la journée?	1	2	3	4
5. Avez-vous besoin d'aide pour manger, vous habiller, faire votre toilette ou aller aux toilettes?	1	2	3	4

**Au cours de la semaine passée:**	Pas du tout	Un peu	Assez	Beaucoup
6. Avez-vous été gênée pour faire votre travail ou vos activités de tous les jours?	1	2	3	4
7. Avez-vous été gênée dans vos activités de loisirs?	1	2	3	4
8. Avez-vous eu le souffle court?	1	2	3	4
9. Avez-vous eu mal?	1	2	3	4
10. Avez-vous eu besoin de repos?	1	2	3	4
11. Avez-vous eu des difficultés pour dormir?	1	2	3	4
12. Vous êtes-vous sentie faible?	1	2	3	4
13. Avez-vous manqué d'appétit?	1	2	3	4
14. Avez-vous eu des nausées (mal au coeur)?	1	2	3	4
15. Avez-vous vomi?	1	2	3	4

Passez à la page suivante S.V.P.

FRENCH NEUTRAL

Au cours de la semaine passée:	Pas du tout	Un peu	Assez	Beaucoup
16. Avez-vous été constipé(e)?	1	2	3	4

Centre Number□□□□□□□□□□ Patient Inclusion Number ⎣—⊥—⊥—⎦

Date of completion of the questionnaire ⎣—⊥—⎦⎣—⊥—⎦⎣—⊥—⊥—⊥—⎦

certaines choses, par exemple, pour lire le journal ou regarder la télévision?	1	2	3	4
21. Vous êtes-vous senti(e) tendu(e)?	1	2	3	4
22. Vous êtes-vous fait du souci?	1	2	3	4
23. Vous êtes-vous senti(e) irritable?	1	2	3	4
24. Vous êtes-vous senti(e) déprimé(e)?	1	2	3	4
25. Avez-vous eu des difficultés pour vous souvenir de certaines choses?	1	2	3	4
26. Votre état physique ou votre traitement médical vous ont-ils gêné(e) dans votre vie familiale?	1	2	3	4
27. Votre état physique ou votre traitement médical vous ont-ils gêné(e) dans vos activités sociales (par exemple, sortir avec des amis, aller au cinéma...)?	1	2	3	4
28. Votre état physique ou votre traitement médical vous ont-ils causé des problèmes financiers?	1	2	3	4

Pour les questions suivantes, veuillez répondre en entourant le chiffre entre 1 et 7 qui s'applique le mieux à votre situation

29. Comment évalueriez-vous votre état de santé au cours de la semaine passée?

1 2 3 4 5 6 7

Très mauvais Excellent

30. Comment évalueriez-vous l'ensemble de votre qualité de vie au cours de la semaine passée?

1 2 3 4 5 6 7

Très mauvaise Excellente

© Copyright 1995 EORTC Study Group on Quality of Life. Tous droits réservés Version 3.0.

EORTC QLQ - H&N35

Les patients rapportent parfois les symptômes ou problèmes suivants. Pourriez-vous indiquer, s'il vous plaît, si, <u>durant la semaine passée</u>, vous avez été affecté(e) par l'un de ces symptômes ou problèmes. Entourez, s'il vous plaît, le chiffre qui correspond le mieux à votre situation.

**Au cours de la semaine passée:**	**Pas du tout**	**Un peu**	**Assez**	**Beaucoup**
31. Avez-vous eu mal dans la bouche?	1	2	3	4
32. Avez-vous eu mal à la mâchoire?	1	2	3	4
33. Avez-vous eu des douleurs dans la bouche?	1	2	3	4
34. Avez-vous eu mal à la gorge?	1	2	3	4
35. Avez-vous eu des problèmes en avalant des liquides?	1	2	3	4
36. Avez-vous eu des problèmes en avalant des aliments écrasés?	1	2	3	4
37. Avez-vous eu des problèmes en avalant des aliments solides?	1	2	3	4
38. Vous êtes-vous étouffé(e) en avalant?	1	2	3	4
39. Avez-vous eu des problèmes de dents?	1	2	3	4
40. Avez-vous eu des problèmes à ouvrir largement la bouche?	1	2	3	4
41. Avez-vous eu la bouche sèche?	1	2	3	4
42. Avez-vous eu une salive collante?	1	2	3	4
43. Avez-vous eu des problèmes d'odorat?.	1	2	3	4
44. Avez-vous eu des problèmes de goût?	1	2	3	4
45. Avez-vous toussé?.	1	2	3	4
46. Avez-vous été enroué(e)?	1	2	3	4
47. Vous êtes-vous senti(e) mal?	1	2	3	4
48. Votre apparence vous a-t-elle préoccupé(e)?	1	2	3	4

<u>Passez à la page suivante S.V.P.</u>

Au cours de la semaine passée:	Pas du tout	Un peu	Assez	Beaucoup
49. Avez-vous eu des difficultés à manger?	1	2	3	4
50. Avez-vous eu des difficultés à manger devant votre famille?	1	2	3	4
51. Avez-vous eu des difficultés à manger devant d'autres personnes?	1	2	3	4
52. Avez-vous eu des difficultés à prendre plaisir aux repas?	1	2	3	4
53. Avez-vous eu des difficultés à parler à d'autres personnes?	1	2	3	4
54. Avez-vous eu des difficultés à parler au téléphone?	1	2	3	4
55. Avez-vous eu des difficultés à avoir un contact social avec votre famille?	1	2	3	4
56. Avez-vous eu des difficultés à avoir un contact social avec vos amis?	1	2	3	4
57. Avez-vous eu des difficultés à sortir en public?	1	2	3	4
58. Avez-vous eu des difficultés à avoir un contact physique avec votre famille ou vos amis	1	2	3	4
59. Avez-vous éprouvé moins d'intérêt aux relations sexuelles?	1	2	3	4
60. Avez-vous éprouvé moins de plaisir sexuel?	1	2	3	4

Au cours de la semaine passée:	Non	Oui
61. Avez-vous pris des anti-douleurs?	1	2
62. Avez-vous pris des suppléments nutritionnels (à l'exclusion de vitamines)?	1	2
63. Avez-vous utilisé une sonde d'alimentation?	1	2
64. Avez-vous perdu du poids?	1	2
65. Avez-vous pris du poids?	1	2

© Copyright 1995 EORTC Quality of Life Study Group, version 1.0 All rights reserved. Tous droits réservés. Version française
