# Supplementary material for: Postoperative SBRT and Severe Late Toxic Effects in Early-Stage Oropharyngeal and Oral Cavity Cancers: The STEREOPOSTOP–GORTEC 2017-03 Nonrandomized Clinical Trial
Source: JAMA Netw Open. 2025 Dec 18;8(12):e2549975. doi: 10.1001/jamanetworkopen.2025.49975 (PMC12715650; doi:10.1001/jamanetworkopen.2025.49975)
Supplement: Supplement 2. — eTable. Mandibular Dose-Volume Indices and Osteoradionecrosis Risk eFigure. Evolution of Mean Quality Of Life Scores Over Time, Assessed Using the EORTC QLQ-C30 and QLQ-HN35 Questionnaires [file jamanetwopen-e2549975-s002.pdf]

Supplemental Online Content

Biau J, Sun X, Liem X, et al. Postoperative SBRT and severe late toxic effects in early-stage oropharyngeal and oral cavity cancers: the STEREOPOSTOP–GORTEC 2017-03 nonrandomized clinical trial. *JAMA Netw Open.* 2025;8(12):e2549975. doi:10.1001/jamanetworkopen.2025.49975

eTable. Mandibular Dose-Volume Indices and Osteoradionecrosis Risk

eFigure. Evolution of Mean Quality Of Life Scores Over Time, Assessed Using the EORTC QLQ-C30 and QLQ-HN35 Questionnaires

This supplemental material has been provided by the authors to give readers additional information about their work.

© 2025 Biau J et al. *JAMA Network Open*.

eTable. Mandibular Dose-Volume Indices and Osteoradionecrosis Risk

	**Grade 3 ORN**	**No Grade 3 ORN**	p-value
**N° events**	7/89 (13.6%)	82/89 (86.4%)	
**CK - VMAT**			
n	4/22 – 3/77	18/22 – 64/77	
% (95%CI)	18% (5.2-40.3) – 4% (0.9-12.4)		0.06
**Mandible**			
V29	21.2 ± 15.2	13.2 ± 10.2	0.17
V32	16.9 ± 14.6	9.6 ± 8.7	0.21
D2%	36.1± 2.6	34.3 ± 4.0	0.52
Dmax	39.2 ± 3.0	38.2 ± 3.9	0.41

Dosimetric values are mean ± standard deviation. Vx denotes the percentage of mandibular volume receiving at least x Gy. D2% is the dose to the hottest 2% of the mandible (near-maximum dose), and Dmax is the maximum point dose. ORN indicates osteoradionecrosis. The small ORN sample (n=7) limits statistical power.

© 2025 Biau J et al. *JAMA Network Open*.

<u>eFigure.</u> Evolution of Mean Quality Of Life Scores Over Time, Assessed Using the EORTC QLQ-C30 and QLQ-HN35 Questionnaires

Higher scores on the **QLQ-C30 Functional** and **Global Health** scales indicate better functioning and overall health, whereas higher scores on the **QLQ-C30 Symptom** and **QLQ-HN35** scales reflect a greater symptom burden.

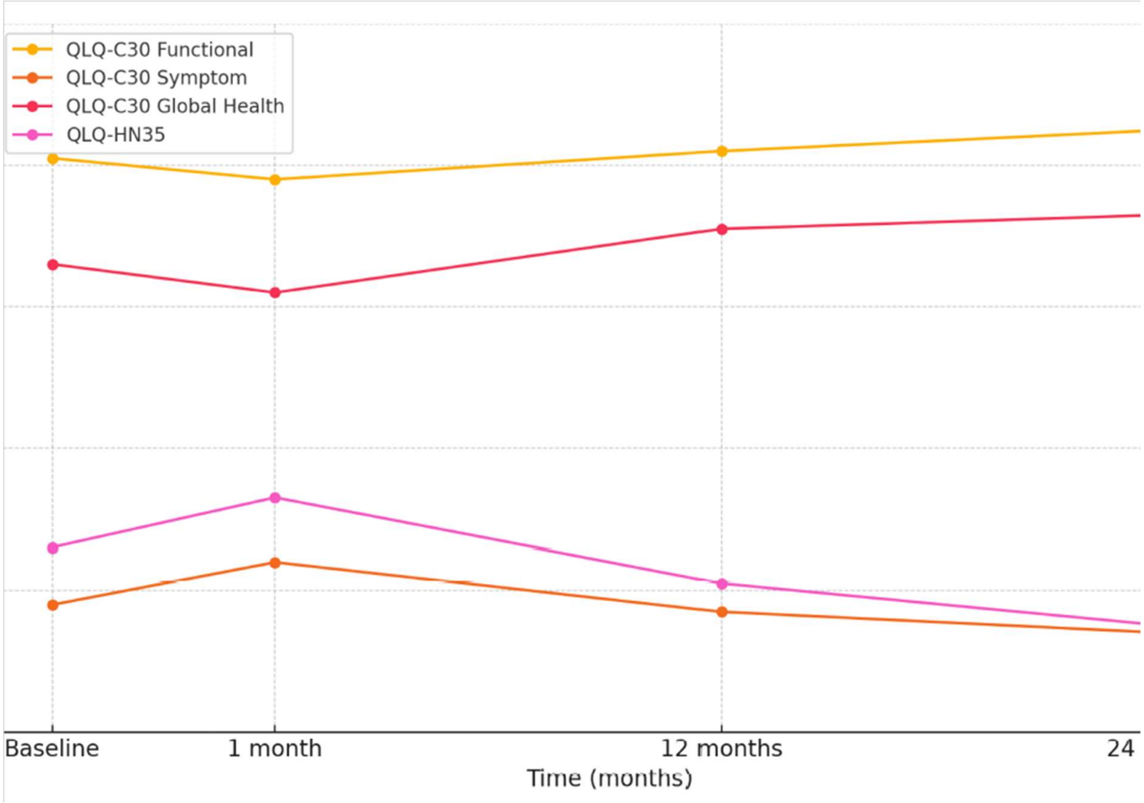

© 2025 Biau J et al. *JAMA Network Open*.
