# Supplementary material for: Postoperative SBRT and Severe Late Toxic Effects in Early-Stage Oropharyngeal and Oral Cavity Cancers: The STEREOPOSTOP–GORTEC 2017-03 Nonrandomized Clinical Trial
Source: JAMA Netw Open. 2025 Dec 18;8(12):e2549975. doi: 10.1001/jamanetworkopen.2025.49975 (PMC12715650; doi:10.1001/jamanetworkopen.2025.49975)
Supplement: Supplement 3. — Data Sharing Statement [file jamanetwopen-e2549975-s003.pdf]

Data Sharing Statement

Biau. Postoperative SBRT and Severe Late Toxic Effects in Early-Stage Oropharyngeal and Oral Cavity Cancers. *JAMA Netw Open*. Published December 17, 2025. doi:10.1001/jamanetworkopen.2025.49975

Data
Additional Information: NCT03401840
Data available: No

Additional Information
Explanation for why data not available: Data can be shared upon request
